# Synthesis, anticancer activity, and molecular docking of half-sandwich iron(II) cyclopentadienyl complexes with maleimide and phosphine or phosphite ligands

**DOI:** 10.1038/s41598-024-56339-0

**Published:** 2024-03-07

**Authors:** Sujoy Das, Marcelina Strachanowska, Piotr Wadowski, Michał Juszczak, Paulina Tokarz, Aneta Kosińska, Marcin Palusiak, Agnieszka J. Rybarczyk-Pirek, Kinga Wzgarda-Raj, Saranya Vasudevan, Arkadiusz Chworos, Katarzyna Woźniak, Bogna Rudolf

**Affiliations:** 1 Department of Organic Chemistry, University of Lodz, Faculty of Chemistry, Tamka 12, 91-403 Lodz, Poland; 2 Department of Molecular Genetics, University of Lodz, Faculty of Biology and Environmental Protection, Pomorska 141/143, 90-236 Lodz, Poland; 3 Department of Physical Chemistry, University of Lodz, Faculty of Chemistry, Pomorska 163/165, 90-236 Lodz, Poland; 4 Centre of Molecular and Macromolecular Studies, Polish Academy of Sciences, Sienkiewicza 112, 90-363 Lodz, Poland

**Keywords:** Biochemistry, Cancer, Chemical biology, Drug discovery, Medicinal chemistry, Chemical synthesis

## Abstract

In these studies, we designed and investigated the potential anticancer activity of five iron(II) cyclopentadienyl complexes bearing different phosphine and phosphite ligands. All complexes were characterized with spectroscopic analysis viz. NMR, FT–IR, ESI–MS, UV–Vis, fluorescence, XRD (for four complexes) and elemental analyses. For biological studies, we used three types of cells—normal peripheral blood mononuclear (PBM) cells, leukemic HL-60 cells and non-small-cell lung cancer A549 cells. We evaluated cell viability and DNA damage after cell incubation with these complexes. We observed that all iron(II) complexes were more cytotoxic for HL-60 cells than for A549 cells. The complex CpFe(CO)(P(OPh)_3_)(η^1^-*N*-maleimidato) **3b** was the most cytotoxic with IC_50_ = 9.09 µM in HL-60 cells, IC_50_ = 19.16 µM in A549 and IC_50_ = 5.80 µM in PBM cells. The complex CpFe(CO)(P(Fu)_3_)(η^1^-*N*-maleimidato) **2b** was cytotoxic only for both cancer cell lines, with IC_50_ = 10.03 µM in HL-60 cells and IC_50_ = 73.54 µM in A549 cells. We also found the genotoxic potential of the complex **2b** in both types of cancer cells. However, the complex CpFe(CO)_2_(η^1^-*N*-maleimidato) **1** which we studied previously, was much more genotoxic than complex **2b**, especially for A549 cells. The plasmid relaxation assay showed that iron(II) complexes do not induce strand breaks in fully paired ds-DNA. The DNA titration experiment showed no intercalation of complex **2b** into DNA. Molecular docking revealed however that complexes CpFe(CO)(PPh_3_) (η^1^-*N*-maleimidato) **2a**, **2b**, **3b** and CpFe(CO)(P(O*i*Pr)_3_)(η^1^-*N*-maleimidato) **3c** have the greatest potential to bind to mismatched DNA. Our studies demonstrated that the iron(II) complex **1** and **2b** are the most interesting compounds in terms of selective cytotoxic action against cancer cells. However, the cellular mechanism of their anticancer activity requires further research.

## Introduction

The discovery of a simple coordination compound *cis*-PtCl_2_(NH_3_)_2_, known as cisplatin and CDDP, with therapeutic potential against a broad spectrum of solid tumors in 1969 was the landmark for the design and development of organometallic compounds as anticancer drug candidates^1^. Further research led to the development of second- and third generation of platinum FDA-approved drugs, namely, carboplatin and oxaliplatin. Moreover, three other cisplatin derivatives (nedaplatin, lobaplatin and heptaplatin) have been approved regionally in China, Japan and South Korea, respectively. The widespread use of platinum drugs is estimated to reach half of all cancer patients who require chemotherapy^2^. Despite the prevalence of platinum drugs in chemotherapeutic regimens, their usage is limited due to intrinsic or acquired drug resistance and serious systemic toxicity including hepatotoxicity, nephrotoxicity, neurotoxicity, and ototoxicity^3^. To overcome the abovementioned drawbacks associated with Pt-based drugs, several strategies have been exploited in the design of small-molecule anticancer metallocompounds. A common approach is the exploration of complexes based on alternative transition metals. In particular, the complexes with metals of group 8, 9 and 10 have been investigated leading to an emergence of a library of non-platinum metal complexes designed as putative anticancer agents.

DNA is the main target of many anticancer drugs. These drugs often induce various damage mechanisms to DNA which inhibit replication and transcription in rapidly dividing cancer cells, leading to their death. For example, cisplatin mainly forms intrastrand DNA adducts, and has a well-documented selectivity for adjacent GG dinucleotide sequences (60–65%) over AG sequences (20–25%). Due to their redox potential and multiple oxidation states, metal complexes (including Pt drugs) can generate reactive oxygen and nitrogen radicals which can also cause DNA damage via base modifications and DNA strand breaks^4^.

Among the transition metal complexes, ruthenium complexes have attracted considerable attention as non-platinum anticancer drug candidates^5^. The most prominent representatives of Ru-based complexes include NAMI-A^6^, KP1019^7^, NKP1339 (IT-139; BOLD-100)^8^ and TLD1443^9^ which have entered clinical trials. Although NAMI-A has passed phase I clinical trials, the phase II was prematurely terminated due to its limited efficacy^6^. The KP1019^7^ and NKP1339^8^ successfully completed phase I trials and TLD-1433 is currently under phase II clinical trial (NCT03945162).

Apart from ruthenium complexes, an increasing interest in anticancer properties of iron complexes has recently been observed^10–12^. The development of iron-based drugs is an expected trend since iron naturally occurs in human organism and thus has lower intrinsic toxicity. The discovery of cytotoxic properties of ferrocene and its oxidized form ferrocenium in 1984, was a groundwork for the development of Fe-based putative anticancer agents^13,14^. The seminal work by Köpf, Köpf-Maier and Neuse laid the foundation for the subsequent research on iron-containing cytotoxic agents which was mainly focused on ferrocene derivatives. One example is ferrocifen which contains ferrocene moiety bound to hydroxytamoxifen—a selective oestrogen receptor modulator used in chemotherapy regiments for breast cancer^15^. The main obstacle hindering entrance of ferrocene derivatives into clinical trial is their poor bioavailability^13^. The half-sandwich cyclopentadienyl-iron complexes overcome this drawback since the structure allows for the attachment of three ligands influencing the final properties of a compound.

**Figure 1:**
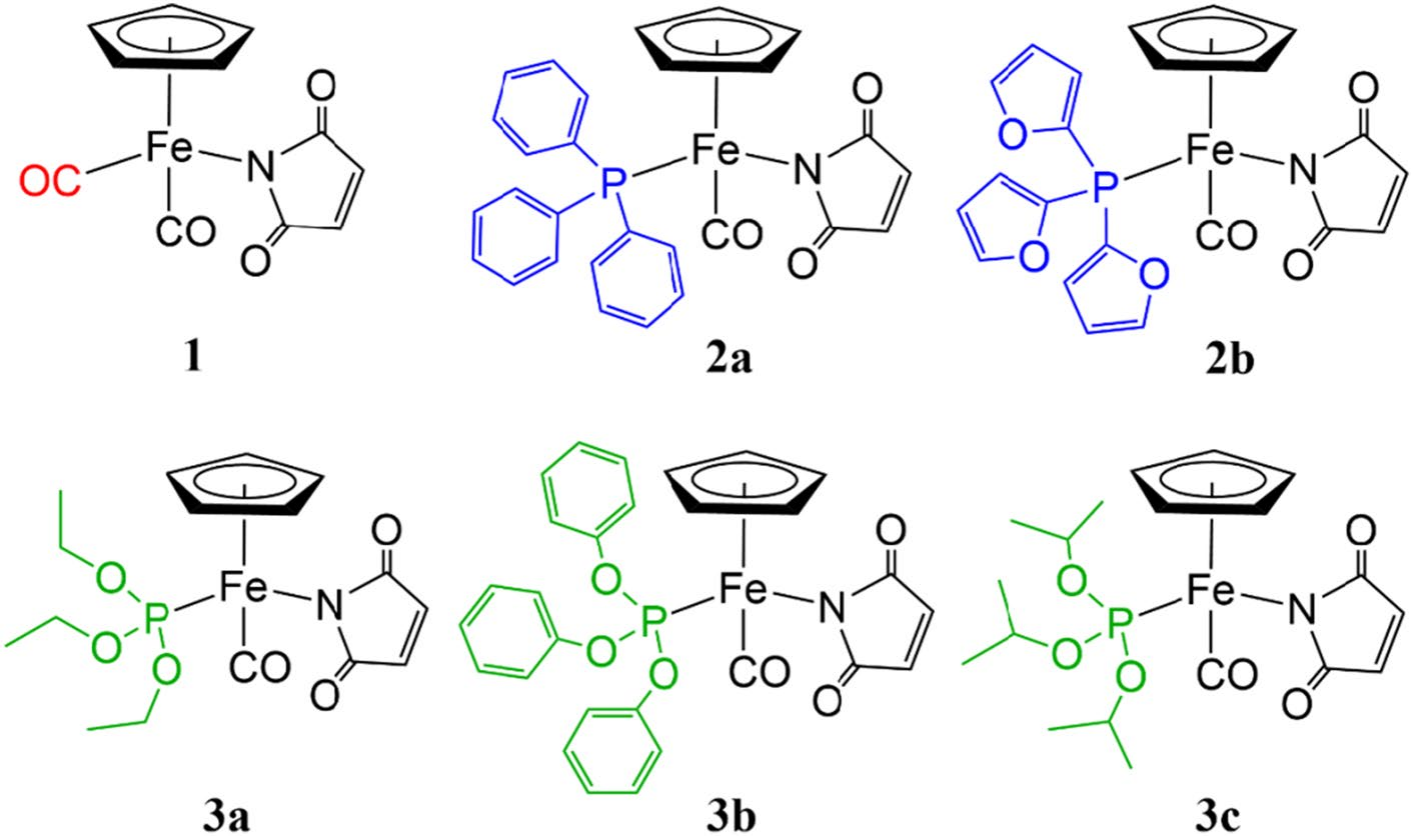
The structures of the half-sandwich complexes **1**, **2a,b** and **3a–c**.

The encouraging results obtained by our group^16,17^ and others ^18–23^ with Ru(*η*^5^-cyclopentadienyl) piano stool complexes led us to the design of five Fe(η^5^-cyclopentadienyl)-compounds as anticancer drug candidates. The complexes have been intentionally functionalized with ligands enhancing anticancer properties including maleimidato, carbonyl, phosphine and phosphite moieties (Fig. 1). All these complexes (**2a**, **2b**, **3a**, **3b** and **3c**) were derivatized from complex **1** in visible light induced CO ligand exchange process. Triphenylphosphine, tri(2-furyl)phosphine, triethyl phosphite, triphenyl phosphite and triisopropyl phosphite have been chosen for the study. The NMR (^1^H, ^31^P, ^13^C), FT-IR, ESI–MS, UV–Vis, fluorescence, XRD and elemental studies were executed for the characterization and structural analysis of these complexes.

The cytotoxic potential of these complexes was analyzed in peripheral blood mononuclear (PBM) cells as normal cells, and two human cancer cell lines—leukemic HL-60 cells and non-small cell lung cancer A549 cells. To study anticancer potential of iron(II) complexes, we selected those complexes that were the most cytotoxic for cancer cells and were less cytotoxic for normal PBM cells, comparing IC_50_ doses. We evaluated the ability of selected iron(II) complexes to induce DNA damage. We also used the plasmid relaxation assay, DFT studies and docking studies to determine the potential of iron(II) complexes to directly damage DNA.

## Results and discussion

### Synthesis of complexes 1, 2a,b and 3a–c

The CpFe(CO)_2_(η^1^-*N*-maleimidato) **1** was obtained following previously described photochemical reaction of CpFe(CO)_2_I with maleimide in presence of diisopropylamine^24^.

**Scheme 1:**
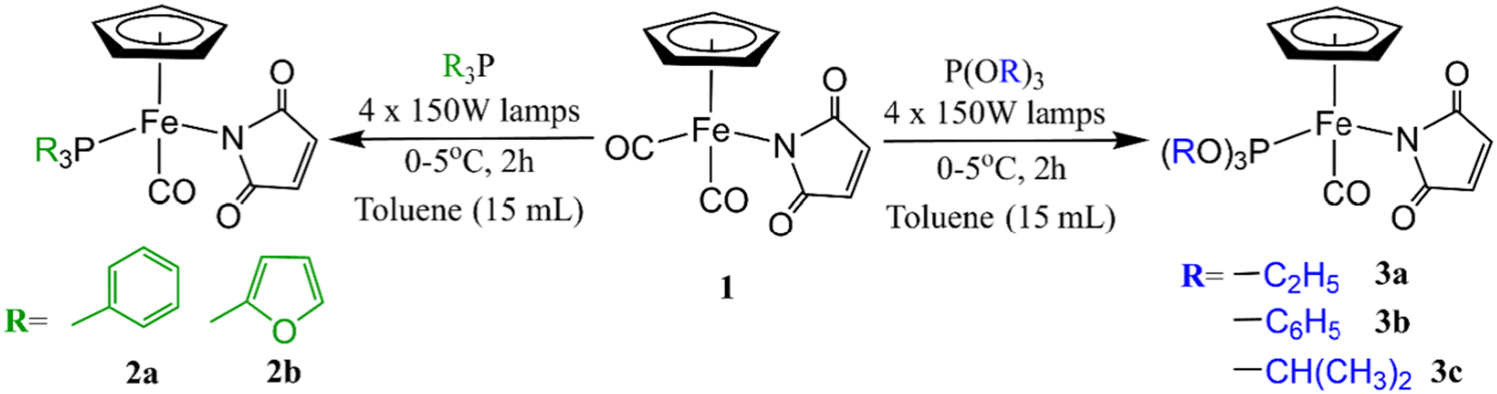
Synthesis of complexes **2a,b** and **3a–c**.

To synthesize complexes **2a,b** and **3a–c**, we modified photochemical ligand exchange reaction of CO by phosphine/phosphite of complex **1** (Scheme 1). A similar synthetic procedure for **2a** has been previously reported by our group where **1** was irradiated with triphenylphosphine by visible light to produce CpFe(CO)(PPh_3_)(η^1^-*N-*maleimidato) in benzene^25^. Here, we replace benzene with toluene to obtain **2a,b** and **3a–c**.

Complex **1** was irradiated with four 150W tungsten lamps in the presence of preferred phosphines (triphenylphosphine, tri(2-furyl)phosphine) or phosphites (triethyl phosphite, triphenyl phosphite and triisopropyl phosphite) under argon to obtain complexes **2a,b** and **3a–c** respectively (Scheme 1).

The crude products were purified by column chromatography and were subsequently characterised by different spectroscopic methods (Figs. S3–S22). Single crystals of **2a**, **2b, 3b** and **3c** were analysed using X-ray diffraction method.

In our previous studies it was evident from the ^31^P NMR analysis that the phosphorus atoms of phosphine and phosphite ligands are being deshielded upon reaction with (η^5^-cyclopentadienyl) ruthenium(II) dicarbonyl maleimidato complex^16^. For this reason, peaks for the ^31^P were found to be shifted downfield in complexes compared to the corresponding ligands. Similar phenomenon has also been observed in iron(II) complexes **2a**,**b** and **3a**–**c** where the degree of downfield shift of ^31^P signal is higher than that of the Ru(II) complex with same ligands as shown in Table 1.

**Table 1:** ^31^P NMR analysis: comparison of the chemical shifts of the phosphines/phosphites, iron and ruthenium complexes **2a,b** and **3a–c**.

Phosphine/phosphites	^31^P NMR (ppm)	Fe-complexes	^31^P NMR (ppm)	Ru-complexes^16^	^31^P NMR (ppm)
P(Ph)_3_	− 5.26	**2a**	75.27	**2a**	56.940
P(Fu)_3_	− 77.00	**2b**	29.25	**2b**	4.797
P(OEt)_3_	139.10	**3a**	175.37	**3a**	148.307
P(OPh)_3_	128.00	**3b**	168.28	**3b**	140.483
P(OiPr)_3_	139.49	**3c**	170.93	**3c**	144.502

The ^1^H and ^13^C NMR spectra of complexes **2a,b** and **3a–c** corroborate to the proposed formulas. The Cp protons of **2a,b** and **3a–c** were shifted upfield compared to the dicarbonyl complex **1**. The most shifted was the Cp signal of complex **3b** bearing the triphenyl phosphite ligand (4.61 ppm for **3b** and 5.05 ppm for **1**). The presence of phosphine or phosphite ligand at the complex has also affected the position of the olefinic protons of maleimide ligand, which was shifted upfield in complexes **2a,b** and **3a–c**. The olefinic protons of **2a** are shifted upfield by 0.45 ppm as compared to those of **1**, which is highest for the corresponding signal among all other complexes.

The ^13^C NMR spectra show the expected signals in the appropriate regions. The olefinic carbons (C=C) were noted around 137 ppm and the imide carbon (C=O) around 185 ppm. The C_C≡O_ resonating at 221–218 ppm like in similar half-sandwich carbonyl compounds we had studied previously. This signal has been shifted downfield by 6–9 ppm compared to that of **1** in all the complexes, probably due to the deshielding effect by phosphorus atom. The C_C≡O_ signal is splitted (d) as a result of the coupling with the phosphorus atom.

No significant change in the signals from the phosphines and phosphites have been observed in the complexes corresponding to the ligands in both ^1^H and ^13^C NMR spectra. Hence, it can be said that the coordination with Fe(II) atom does not affect much the structure of the ligand part in the complexes **2a**,**b** and **3a**–**c**.

The absorbance and emission spectra of all complexes were recorded in chloroform. For example, compound **2a** and **2b** shows emission peaks at 345 nm and 347 nm, with the excitation at 300 nm and 280 nm respectively. It is observed that the characteristic emission peaks of most of the phosphine/phosphite ligands have been suppressed after formation of the complex with iron (Figs. S24 and S25).

### Crystal structure description

**Figure 2:**
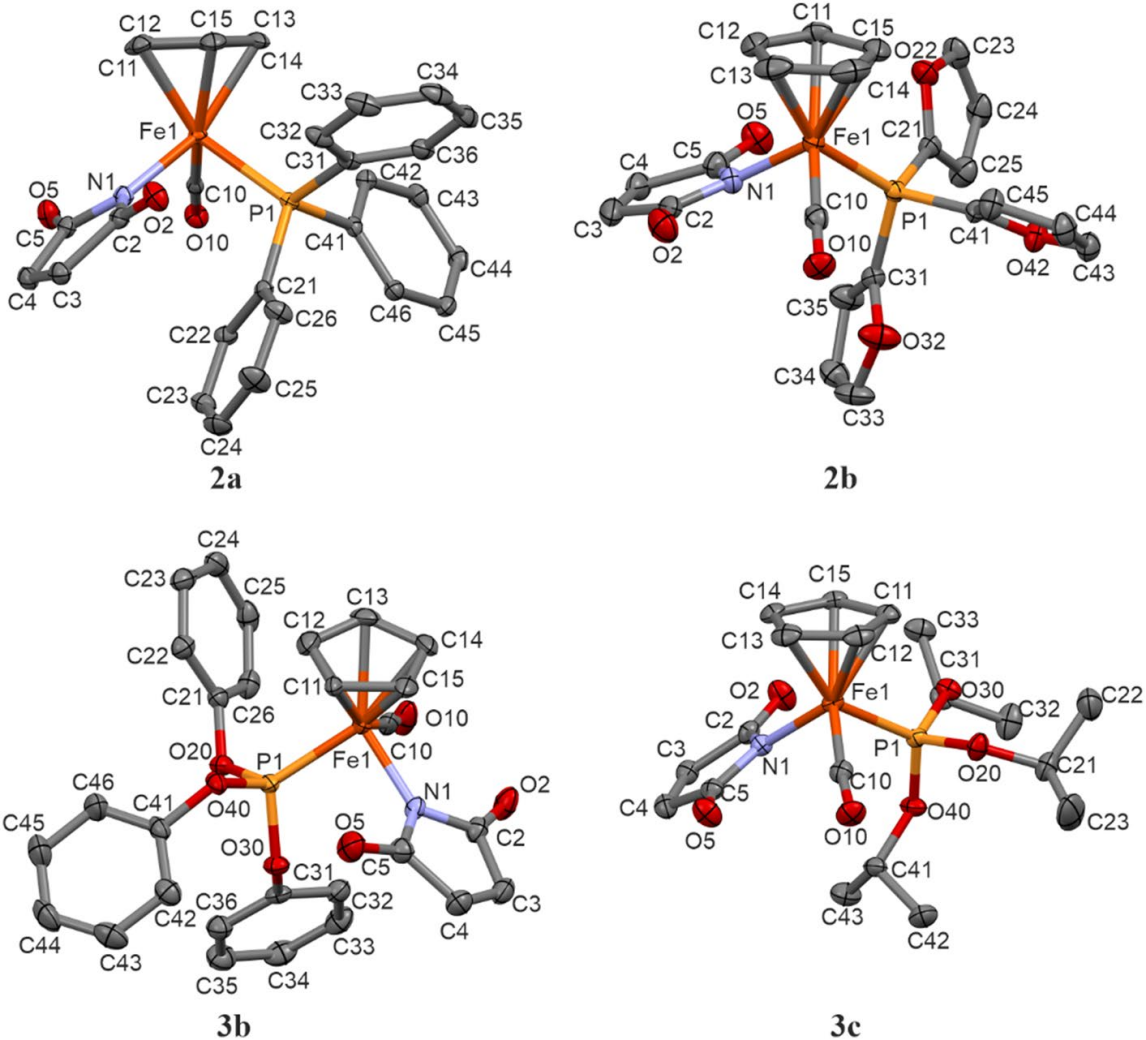
Molecular structures of the investigated compounds with atom labelling scheme: **2a**, **2b**, **3b** and **3c**. Displacement ellipsoid are drawn with 40% probability level, hydrogen atoms and minor disorder component of **3b** are omitted for clarity.

The results of crystal structure determination and molecular structures with corresponding atom labelling schemes are presented in Table 2 and Fig. 2. As seen, in all the cases iron Fe1 atom is bonded to cyclopentadienyl ring (C11–C12–C13–C14–C15), carbonyl ligand (C10–O10), nitrogen atom N1 of maleimidato ligand and phosphorus P1 of phosphine or phosphite ligand.

**Table 2:** Details of X-ray diffraction measurements and crystal structure determination.

	**2a**	**2b**	**3b**	**3c**
Molecular formula	C_28_H_22_FeNO_3_P	C_22_H_16_FeNO_6_P	C_28_H_22_FeNO_6_P	C_19_H_28_FeNO_6_P
M ([g mol^−1^)	507.28	477.18	555.28	453.24
Crystal system	triclinic	orthorhombic	monoclinic	monoclinic
space group	P*-1*	P*na2*_*1*_	C*2/c*	P*2*_*1*_*/c*
a [Å]	8.04065(10)	13.2089(2)	16.8587(2)	15.32170(10)
b [Å]	9.87234(13)	10.55910(10)	8.62090(10)	8.89300(10)
c [Å]	15.02351(17)	14.1422(2)	35.2763(3)	15.83020(10)
α [°]	77.1258(10)	90	90	90
β [°]	87.8415(10)	90	94.2030(10)	94.1040(10)
γ [°]	79.6894(10)	90	90	90
V [Å^3^]	1143.81(2)	1972.47(4)	5113.17(10)	2151.43(3)
Z	2	4	8	4
Crystal size (mm^3^)	0.044 × 0.104 × 0.208	0.106 × 0.068 × 0.045	0.196 × 0.058 × 0.043	0.174 × 0.104 × 0.021
*d*_*x*_ (mg m^−3^)/μ (mm^−1^)	1.473/0.761	1.607/7.273	1.443/5.696	1.399/6.620
F(000)	524.0	976.0	2288.0	952.0
λ [Å], T [K]	Mo Kα, 100(2)	Cu Kα, 100(2)	Cu Kα, 100(2)	Cu Kα, 100(2)
2θ range [°]	5.15–51.996	10.456–135.994	5.024–153.754	5.784–153.782
Index ranges	− 9 ≤ h ≤ 9 − 12 ≤ k ≤ 12 − 18 ≤ l ≤ 18	− 15 ≤ h ≤ 15 − 12 ≤ k ≤ 12 − 16 ≤ l ≤ 16	− 20 ≤ h ≤ 21 − 10 ≤ k ≤ 10 − 42 ≤ l ≤ 44	− 18 ≤ h ≤ 19 − 11 ≤ k ≤ 9 − 19 ≤ l ≤ 19
Data collected/unique	52,838/4483 R_int_ = 0.0371	21,719/3565 R_int_ = 0.0440	29,598/5124 R_int_ = 0.0260	33,257/4378 R_int_ = 0.0323
data/restraints/parameters	4483/0/307	3565/1/280	5124/15/381	4378/0/260
GooF on *F*^*2*^	1.075	1.032	1.042	1.033
*R*/*wR*^*2*^ [I > 2σ (I)]	R_1_ = 0.0234 wR_2_ = 0.0606	R_1_ = 0.0357 wR_2_ = 0.0928	R_1_ = 0.0253 wR_2_ = 0.0635	R_1_ = 0.0269 wR_2_ = 0.0658
*R*/*wR*^*2*^ (all data)	R_1_ = 0.0250 wR_2_ = 0.0612	R_1_ = 0.0366 wR_2_ = 0.0934	R_1_ = 0.0267 wR_2_ = 0.0642	R_1_ = 0.0279 wR_2_ = 0.0664
Δρ_min_/Δρ_max_ [e∙Å^−3^]	0.37/− 0.27	1.20/− 0.23	0.29/− 0.40	0.45/− 0.28
Flack parameter	–	0.002(4)	–	–
CCDC number	2,280,168	2,280,165	2,280,167	2,280,166

All the investigated compounds can be classified as half-sandwich complexes with cyclopentadienyl moiety arranged on the opposite side of central Fe1 atom in respect to the three other ligands. Among the coordination bonds the longest are Fe1-P1 (from 2.144(4)Å to 2.221(2)Å) and a little shorter, of length about 1.77 Å, Fe1-N1 to maleimidato ligands (compare Table S1). Bond distances between iron and carbon atoms of carbonyl ligand (Fe1-C10) are changing from 1.754(2)Å to 1.773(5)Å. In turn, the shortest bonds are observed to cyclopentadienyl moiety with Fe1-Cg1 distance of about 1.72 Å, excluding data for the disordered structure **3b** (Cg1 corresponds to the centre of gravity of cyclopentadienyl ring).

The valence angles around Fe1 and between N1 (maleimidato), P1 (phosphine/phosphite) and C10 (carbonyl) atoms are very close to 90°. It may be stated, that Fe1-N1, Fe1-P1 and Fe1-O10 bonds are perpendicular and cross each other in the position of the central iron atom. In turn, the valence angles to the cyclopentadienyl ring (Cg1) are all above 120°. All these values of geometric parameters are in agreement with the known for half-sandwich compounds “piano-stool” conformation^17^.

Assuming that cyclopentadienyl is treated as a single ligand, we may state that coordination number for iron is equal to four and call the coordination sphere in molecules of all compounds a strongly distorted tetrahedron. For such tetrahedral four-coordinated Fe1 atom, similarly like for asymmetric sp^3^ carbon atom, there can be indicated as a formal configuration. Using the Cahn-Ingold-Prelog^26^ rules, the coordinative ligands can be listed in the following order: I—phosphine/phosphite, II—maleimidato, III—carbonyl, IV—cyclopentadienyl, and hence two opposite conformations (corresponding to *R* and *S* stereoisomers) can be indicated.

Compounds **2a**, **3b** and **3c** crystallize in centrosymmetric space groups of triclinic or monoclinic system. In such cases, because of the presence of inversion centre, there exist two opposite conformers in the crystalline state. Interestingly, compound **2b** crystallizes in the non-centrosymmetric orthorhombic P*na2*_*1*_ space group, nevertheless, there are also observed *R* and *S* stereoisomers due to the presence of glide mirror planes (*n*_*x*_ and *a*_*y*_) among space group symmetry elements. This is in contrast to analogical ruthenium complex which due to spontaneous resolution during crystallization shows P*2*_*1*_*2*_*1*_*2*_*1*_ space group symmetry and the reported crystal forms only one *R* stereoisomer^16^.

**Figure 3:**
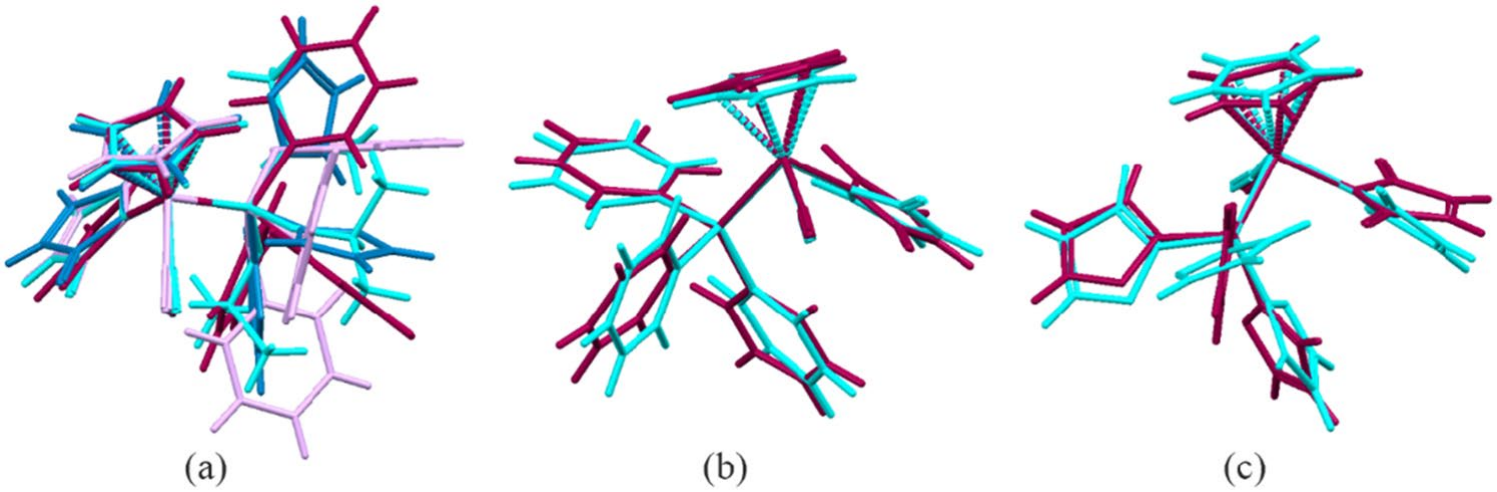
Comparison of molecular conformations observed in crystal structures: (**a**) **2a** (purple), **2b** (blue), **3b** (lilac) and **3c** (cyan); analogic complexes of ruthenium (cyan) and iron (purple)—**2a** (**b**) and **2b** (**c**).

Figure 3 presents overlay and comparison of molecular conformations including both currently investigated iron complexes and previously published results of their ruthenium analogues.

For comparison of the overall molecular geometry the same isomers have been considered in overlapping procedure (Table S1—compare torsion angles). As seen, there are no significant differences between iron–ruthenium pairs of compounds (see Fig. 3b,c). Sligh conformational changes result from rotation about P1-C21/C31/C41 bonds of aromatic rings. In turn, larger differences are seen in a group of iron complexes, in particular with phosphite ligands. This is due to the higher flexibility of sp^3^ oxygen O20/O30/O40 atoms (compare Figs. 2 and 3a).

Even though, molecular conformations in the investigated structures are in general rather similar, their final crystal packings vary significantly because of different crystal systems and space groups. This is mainly the result of various size, shape and electron donor–acceptor properties of phosphoroorganic ligands. However, the one thing is common to all structures—they belong to the class of molecular crystals of structure stabilized by non-covalent interactions. In the observed relatively weak C–H…O hydrogen bonds oxygen atoms from carbonyl groups are taking part as proton acceptors. Some molecular complexes linked by these bonds are shown in the Fig. S23 and the corresponding interaction geometric parameters are gathered in the Table S2.

### DFT studies

**Figure 4:**
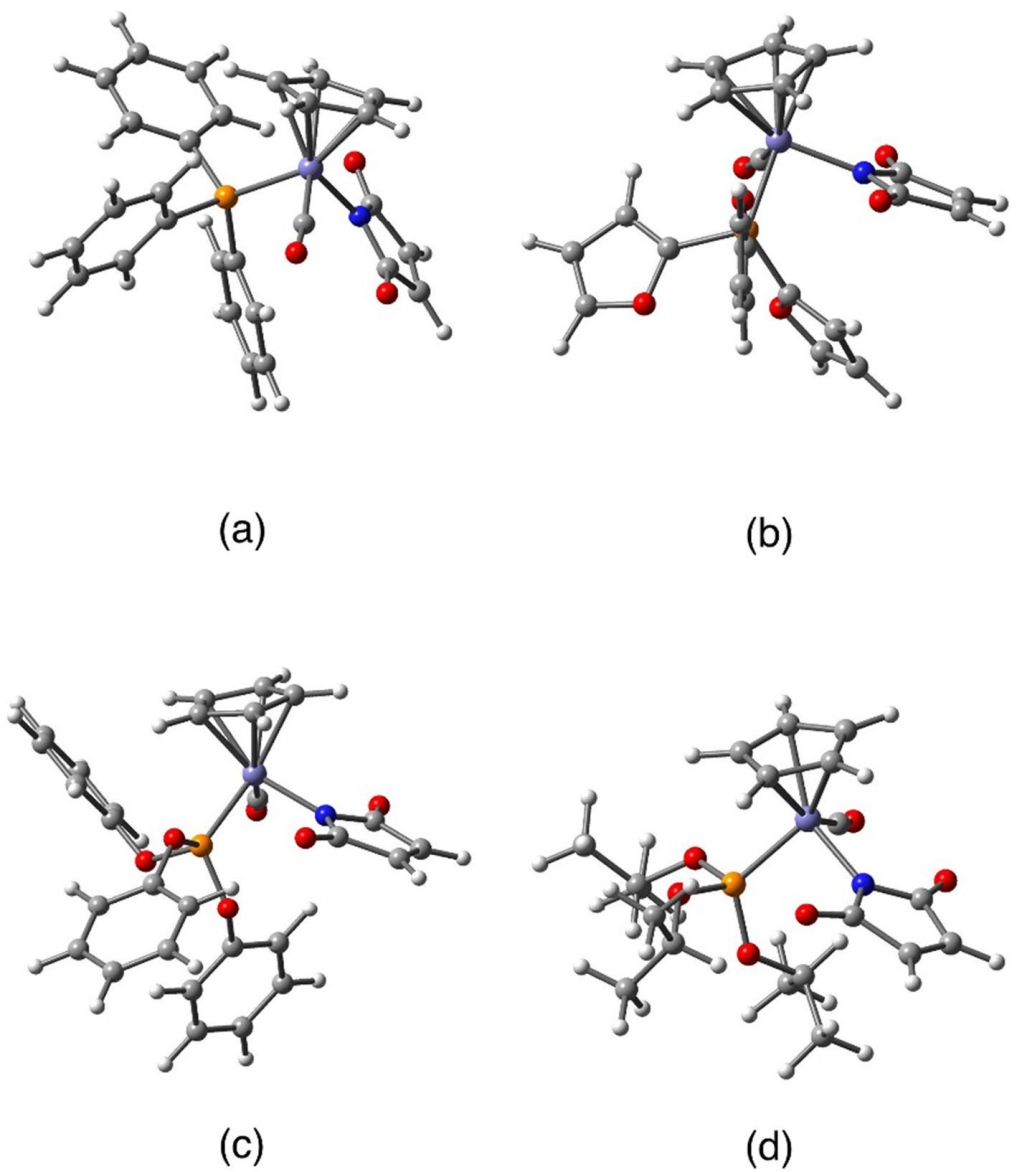
Optimized geometries of model system chosen for computational analysis; **2a** (**a**), **2b**, (**b**), **3b**, (**c**), and **3c**, (**d**).

In order to gain insight into the reactivity of the investigated compound, a frontier orbital analysis was conducted using DFT calculations. For this purpose, a full optimization was performed on isolated molecules, utilizing the wB97XD^27^ functional in conjunction with the def2TZVP basis set^28^, implemented in the Gaussian16 suite^29^. The molecular structure obtained from X-ray data served as the starting point for geometry optimization. The HOMO/LUMO energies were calculated for the optimized geometries and are presented in Table 3, optimized geometries of studied compounds are presented in Fig. 4.

**Table 3:** The HOMO/LUMO energies calculated for the optimized geometries for complexes **2a,b** and **3b,c**.

	**2a**	**2b**	**3b**	**3c**
HOMO [hartree]	− 0.290	− 0.295	− 0.305	− 0.295
LUMO [hartree]	0.005	0.007	0.001	− 0.000
HOMO/LUMO gap [hartree]	0.296	0.302	0.306	0.294
HOMO/LUMO gap [kcal/mol]	185.4	189.6	192.1	184.6
Relative HOMO/LUMO gap [kcal/mol]	0.8	5.0	7.5	0.0

Based on the DFT calculations, the HOMO/LUMO gap for all four derivatives is approximately 0.3 hartrees. The smallest gap was observed for the **3c** molecule with similar value for **2a**, differing by only about 1 kcal/mol. In the case of the other two compounds, the HOMO/LUMO gap is relatively higher, with the highest value found for **3b** being above 7 kcal/mol. Therefore, based on the thermodynamic properties of the isolated molecular models, **3c** and **2a** are expected to be the most active, while the other two compounds appear to have lower activity.

### Results of the cytotoxicity study

We examined the viability of cells after 2 h and 24 h incubation with iron(II) complexes using the resazurin reduction assay (Tables S3 and S4, respectively). We observed no change in the viability of PBM cells during short, 2 h, incubation with complexes **1**, **3a** and **3b**. At high concentrations, the complex **2b** presented cytotoxic properties while the complex **3c** increased PBM cells viability which could be explained by the increased activity of the cells after 2 h treatment with the compound. The complex **2a** slightly decreased PBM cell viability at low concentrations (Table S3). In the case of 24 h incubation, we observed a decrease in the viability of PBM cells with the increasing concentrations of all complexes with the exception of complex **3a** (Table S4).

We observed a decrease in the viability of HL-60 cells with increasing concentrations of complexes **1, 2b** and **3a** after 2 h incubation. The complex **2a** caused a slight increase in the viability of these cells. We observed a sharp increase (up to approximately 140%) in the HL-60 cell viability for complexes **3b** and **3c** (Table S3). However, the highest concentration (250 μM) of these compounds led to a sharp decrease in HL-60 cell viability. The viability of HL-60 cells was decreased for all complexes with increasing concentrations after 24 h incubation. Among the studied compounds, the complex **3b** was the most cytotoxic (Table S4).

In the case of A549 cells was observed a steady decrease in cell viability with increasing concentration of complexes **1**, **2a** and **3a** after a short, 2 h incubation (Table S3). We also observed an increased viability (123%) of HL-60 cells after short treatment with complex **2b** at the concentration of 50 μM which later dropped sharply to 40% at 250 μM. Incubation with **3c** resulted in steady rise in the HL-60 cell viability to the point of 134% at 250 μM, and in the case of **3b** no change in the viability was observed. Regarding 24 h incubation, an increase in the viability of A549 cells was observed with increasing concentrations of complexes **2a** and **3a** to 120% and 153%, respectively (Table S4). The complexes **1**, **2b** and **3b** sharply decreased A549 cell viability at higher concentrations, while complex **3c** decreased viability to approximately 69% at concentration of 5 μM and increased viability at higher concentrations.

Then, we determined IC_50_ doses for all iron(II) complexes against the three tested cell types after a 24 h incubation (Table 4). HL-60 cells showed a decrease in the viability with increasing concentrations for all complexes. Complexes **1**, **2b**, **3a** and **3c** were cytotoxic for HL-60 cells while maintaining low cytotoxicity for normal PBM cells. Among studied compounds the most promising against HL-60 cells were complexes **2b** and **3a** (IC_50_ = 10.03 μM and 15.93 μM, respectively) without detected cytotoxicity against PBM cells (IC_50_ > 250 μM). Our studies also indicated that the complex **2b** bearing maleimide and phosphine ligands was selectively cytotoxic for both cancer cell lines, HL-60 cells (IC_50_ = 10.03 µM) and A549 cells (IC_50_ = 73.54 µM) and not for the normal PBM cells (IC_50_ > 250). The selective targeting of cancer cells by complex **2b** is promising and fits into the development of new generation therapeutic agents oriented toward protection of normal cells.

**Table 4:** IC_50_ values for iron(II) complexes measured after 24 h incubation of PBM, HL-60 and A549 cells.

Iron(II) complexes	PBM cells (µM)	HL-60 cells (µM)	A549 cells (µM)
1	105.63	7.69	54.2
2a	61.43	14.84	> 250
2b	> 250	10.03	73.54
3a	> 250	15.93	> 250
3b	5.80	9.09	19.16
3c	104.32	21.44	> 250

Interestingly, the iron(II) complex **2b** was more active to HL-60 cells than the analogous ruthenium compound **2b** CpRu(CO)(PPh_3_) (η^1^-*N*-maleimidato) that we studied previously^15^ (IC_50_ = 10.03 µM for Fe and IC_50_ = 35.64 µM for Ru). It should be noted that the iron complex **2b** is not active to PBM cells (IC_50_ > 250 µM), but the ruthenium complex **2b** was cytotoxic to PBM cells (IC_50_ = 8.48 µM). The iron(II) complex **3a** showed a very similar IC_50_ for HL-60 cells (IC_50_ = 15.93 µM) to the analogous ruthenium(II) complex **3a** (CpRu(CO)(P(OEt)_3_)(η^1^-*N*-maleimidato)) (IC_50_ = 12.25 µM). Both complexes were also non-cytotoxic to normal PBM cells (IC_50_ > 250 µM).

Based on the cytotoxicity results, iron(II) complexes **1**, **2b**, **3a** and **3c** were selected for genotoxic studies involving HL-60 cells. In the case of A549 cells, among iron(II) complexes with limited cytotoxicity against PBM cells (IC_50_ > 250 μM) complex **2b** was the most cytotoxic (IC_50_ = 73.54 μM). The complexes **2a**, **3a** and **3c** did not show cytotoxicity against A549 cells. Although, the complex **1** demonstrated the highest cytotoxic properties among studied compounds against A549 cells (IC_50_ = 54.2 μM), it was also cytotoxic for PBM cells at the concentration twice as high (IC_50_ = 105.63 μM). Thus, complexes **1** and **2b** were selected for further studies on A549 cell line.

### Results of the genotoxicity study

We performed genotoxic assays to investigate the mechanism of cytotoxic properties of iron(II) complexes. The ability to induce DNA damage was examined by both the comet assay and the plasmid relaxation assay. The comet assay allows for the analysis of DNA damage in single cell whereas the plasmid relaxation assay discriminate the genotoxic potential at a molecular level.

We performed alkaline version of the comet assay, which allows for the detection of DNA double and single strand breaks and alkali labile sites, after 2 h incubation of cancer cells with selected iron(II) complexes. We deliberately chosen short incubation period to eliminate the possibility of DNA damage induction as secondary effect e.g., as a result of apoptosis. Additionally, we performed the cell viability assay after 2 h incubation with cells to further exclude this effect.

**Figure 5:**
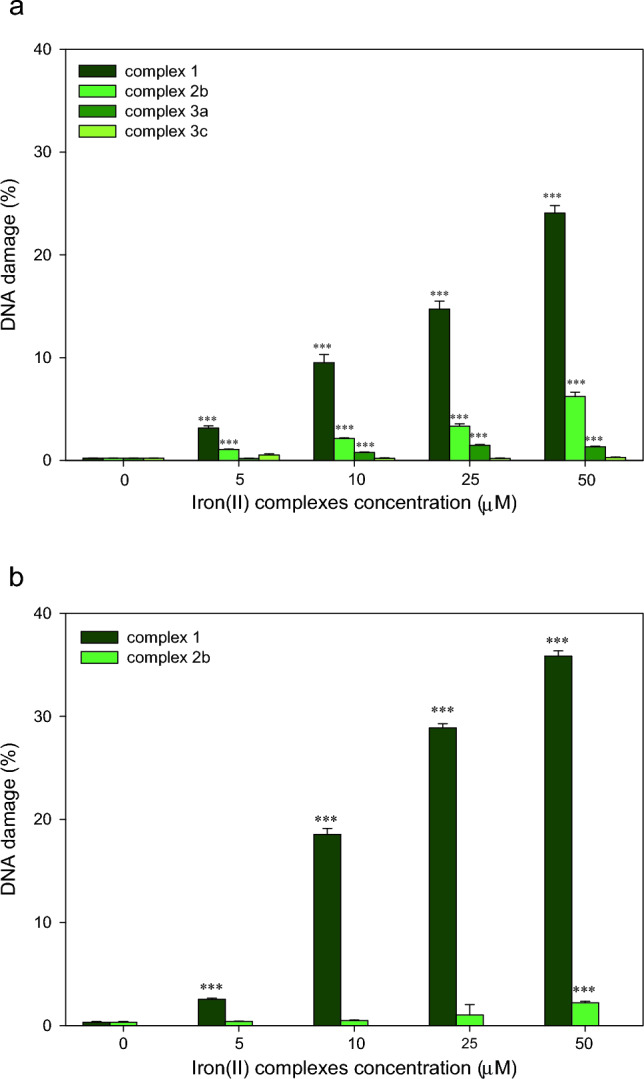
DNA damage in (**a**) HL-60 cells and (**b**) A549 cells incubated for 2 h at 37 °C with selected iron(II) complexes analysed by the alkaline comet assay. The figure shows mean results ± SEM, *n* = 100; ***p < 0.001.

We observed that the complex **1** and **2b** induced DNA damage in HL-60 cells in the concentration range 5–50 μM (p < 0.001) (Fig. 5a). The complex **1** was more genotoxic than complex **2b**, inducing 25% DNA in tail vs. 6% DNA in tail at concentration 50 μM. In the case of complex **3a,** we observed DNA damage at the concentrations of 10, 25 and 50 μM (p < 0.001), however, the percentage of DNA in tail has not increased in a concentration-dependent manner. The DNA damage for complex **3c** was not detected in these conditions.

We did not observe any increase in the level of DNA damage in A549 cells in the range of tested concentrations for complex **2b** with the exception at the concentration of 50 μM (p < 0.001) (Fig. 5b). The percentage of DNA damage in tail was equal to about 2%.

Previously, we studied iron(II) complex **1** (η^5^-C_5_H_5_)Fe(CO)_2_(η^1^-*N*-maleimidato) in HL-60 cells. We have shown that this complex damages DNA and causes a significant increase in the expression of *HO-1* gene, in contrast to the complex of iron(II) with succinimide ligand (η^5^-C_5_H_5_)Fe(CO)_2_(η^1^-*N*-succinimidato). Furthermore, DNA damage induced by complex **1** was not effectively repaired in HL-60 cells^30^.

In recent years, many iron complexes have been synthesized and their biological activity was described. For example, a series of diiron cyclopentadienyl complexes containing bridging vinyliminium ligands against cisplatin sensitive and resistant human ovarian carcinoma (A2780 and A2780cisR) cell lines were investigated^31^. Notable selectivity towards these cancerous cell lines was observed as compared to the non-cancerous 293 T cell line. The anticancer activity of these complexes was associated with the induction of reactive oxygen species (ROS). Moreover, cyclopentadienyl iron complexes with the general formula [CpFe(CO)(PPh_3_)(NCR)]^+^ (NCR = benzonitriles) were tested against breast MDA-MB-231 and colorectal SW480 cancer cells with IC_50_ at low micromolar range. These compounds caused apoptosis, inhibited colony formation and affected cell cytoskeleton organization^32^. Another study evaluated the antiproliferative activity of iron(II) cyclopentadienyl complexes bearing n-heterocyclic carbene ligands in human colorectal (HCT116) and ovarian (A2780) carcinoma cells and in vivo. The complex ([Cp(IMes)Fe(CO)_2_]I) (IMes = 1,3-bis(2,4,6-trimethyl-phenyl)imidazol-2-ylidene) displayed higher than cisplatin cytotoxic activity both in HCT116 and A2789 cells with IC_50_ values in the low micromolar range. Interestingly, this complex decreased the proliferation of colorectal HCT116 cancer cell in vivo while demonstrated low in vivo toxicity as analyzed in zebrafish (*Danio rerio*) xenograft^32^.

The latest studies indicate that iron complexes can inhibit the activity of the multidrug resistance protein ABCB1^33^. The research was carried out using doxorubicin-sensitive cells (Colo205) and doxorubicin-resistant (Colo320) human colon adenocarcinoma cell lines. Compound [CpFe(CO)(PPh_3_) (1-benzylimidazole)](CF_3_SO_3_) was the most active in both cell lines with IC_50_ values of 1.26 ± 0.11 and 2.21 ± 0.26 μM, respectively, being also slightly selective towards cancer cells vs. MRC5 human embryonic fibroblast cell lines. This compound, together with [CpFe(CO)(PPh_3_)(1H-1,3-benzodiazole)](CF_3_SO_3_), was found to display very potent ABCB1 inhibitor activity. [CpFe(CO)(PPh_3_)(1-benzylimidazole)](CF_3_SO_3_) also showed the ability to induce apoptosis. Iron cellular accumulation studies by ICP-MS and ICP-OES methods revealed that cytotoxicity of these complexes was not related to the extent of iron accumulation. On the other hand, [CpFe(CO)(PPh_3_)(1-benzylimidazole)][CF_3_SO_3_] was the only one where iron accumulation was greater in the resistant cell line than in the sensitive one, validating the possible role of ABCB1 inhibition^33^.

**Figure 6:**
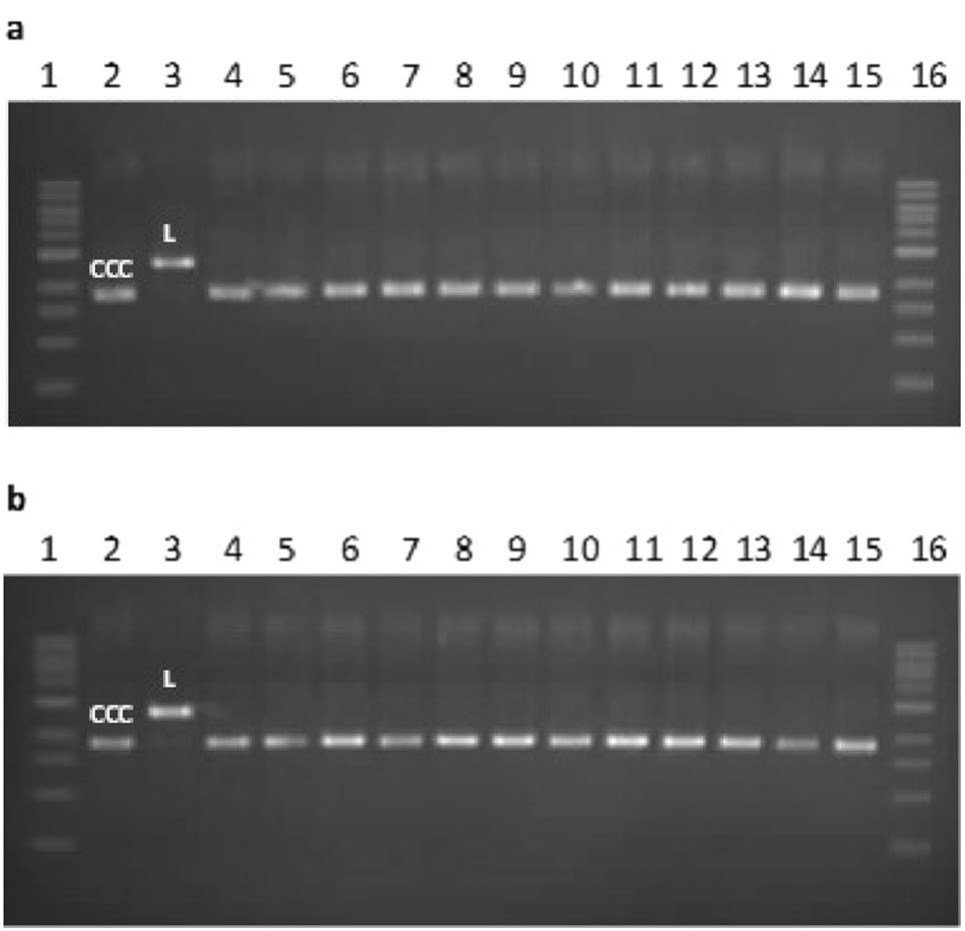
Plasmid relaxation assay. Plasmid pUC19 was incubated for 2 h (**a**) and 24 h (**b**) in 37 °C with iron(II) complexes at concentrations 5 μM and 50 μM, and then was resolved on a 1% agarose gel, stained with ethidium bromide and visualized in UV light. Lines 1 and 16—DNA ladder; line 2—pUC19 plasmid in the supercoiled (CCC) form; line 3—pUC19 plasmid incubated with restrictase Pst*l* in the linear (L) form; line 4 and 5—pUC19 plasmid incubated with complex **1** at 5 μM and 50 μM, respectively; line 6 and 7—pUC19 plasmid incubated with complex **2a** at 5 μM and 50 μM, respectively; line 8 and 9—pUC19 plasmid incubated with complex **2b** at 5 μM and 50 μM, respectively; line 10 and 11—pUC19 plasmid incubated with complex **3a** at 5 μM and 50 μM, respectively; line 12 and 13—pUC19 plasmid incubated with complex **3b** at 5 μM and 50 μM, respectively; line 14 and 15—pUC19 plasmid incubated with complex **3c** at 5 μM and 50 μM, respectively*.*

We also investigated the induction of DNA damage by the iron(II) complexes using the plasmid relaxation assay. We isolated the pUC19 plasmid from the DH5α *E. coli* cells in its supercoiled form (CCC) (Fig. 6, line 2). We treated the pUC19 plasmid with restrictase *Pst*I overnight at 37 °C, obtaining linear (L) form of the plasmid (Fig. 6, line 3). Next, we incubated native plasmid (CCC) with iron(II) complexes at concentrations 5 μM and 50 μM. We observed no degradation of the plasmid after 2 h and 24 h incubation (Fig. 6a,b, respectively). Neither open circular form of pUC19 plasmid (OC) nor the L form of the plasmid was visible. These results suggest that the iron(II) complexes do not cause DNA damage at a molecular level.

Similarly, new dinuclear iron(II) complexes containing iminopyridine ligands based on a methanodibenzodioxocine (DBDOC) backbone did not induce DNA breaks in the plasmid relaxation assay^34^. However, in the presence of H_2_O_2_ these complexes promoted DNA double strand breaks. The Cu^2+^/Zn^2+^/Fe^2+^/Fe^3+^ complexes containing different ligand scaffolds such as phenanthroline, bipyridine, terpyridine, cyclen, hydrazones, triazole, imidazole and protected phenol have been reported to concurrently bind and cleave DNA without any external redox agent(s). In most cases, ROS such as superoxide ions (O_2_^⋅−^), hydroxyl radicals (^⋅^OH), singlet oxygen (^1^O_2_)/singlet oxygen like species or H_2_O_2_ played active roles in DNA cleavage. In some cases, transient metal bound species were also created and were responsible for DNA cleavage. Metal complexes with nucleolytic activity that do not require the participation of additional activating factors are of great interest to researchers because such complexes could be used in anticancer therapy^35^. Unlike the iron(II) complexes studied here, the analogous ruthenium(II) complexes, studied previously, induced DNA breaks *in vitro*^16^.

Cancer cells accumulate and use more iron than normal cells, due to the higher proliferation and DNA synthesis. Furthermore, iron plays a crucial role in the regulation of cell cycle by affecting both the formation and the activity of cyclin proteins (cyclin A, B, D, and E) and cyclin-dependent kinase (CDKs) complexes. Cancer cells overexpress genes involved in the iron metabolism and the iron-sulfur (Fe-S) cluster biogenesis. The initial Fe–S cluster synthesis occurs within the mitochondria; however, the maturation of Fe–S clusters culminating in their ultimate insertion into appropriate cytosolic/nuclear proteins is coordinated by a late-acting cytosolic iron-sulfur assembly (CIA) complex in the cytosol. Several nuclear proteins involved in DNA replication and repair interact with the CIA complex and contain Fe–S clusters necessary for proper enzymatic activity. Moreover, it is currently hypothesized that the late-acting CIA complex regulates the maintenance of genome integrity and is an integral feature of DNA metabolism^36^. Iron metabolism disorders including Fe–S clusters leading to iron accumulation in the cell and lipid peroxidation can cause cell death by ferroptosis^37^.

### Results of the Docking studies

The binding energy analysis suggests the highest DNA binding potential for **2a**, **2b** and **3b** (Table 5 and Fig. S26). This may be linked with the fact that all these compounds are aromatic derivatives. Complex **3b** is toxic to all cell lines, including normal PBM cells (Table 4). Complex **2a** is toxic towards cancerous HL-60 cells, but also towards normal cell line and both compounds (**2a** and **3b**) are phenyl derivatives. Interestingly, **2b** analog is an aromatic derivative, but with furan not phenyl substituents; this seems to be most promising compound expressing toxicity towards cancerous cells (HL-60 and A549), however without detectable cytotoxicity towards normal human blood derived PBM cells (Table 4). What is interesting all tested compounds (**2a**,**b** and **3b,c**) have relatively low binding energy with fully paired DNA fragment (Table 5). However, tested iron(II) compounds bind 3 times stronger with mismatched DNA, where all tested complexes appear to be located at the level of T-T mismatch (Fig. S27a,b). This suggests that when DNA is damaged it can recruit Ru(II)^16^, or for that matter also Fe(II) complexes, which might cause additional DNA degradation and/or alter the DNA repair mechanisms inside cells. These results corroborate with cellular experiments and might explain higher cytotoxicity; however, additional tests are necessary to prove this mechanism.

**Table 5:** Docking score of DNA and mismatched DNA with iron(II) complexes with maleimide and phosphine or phosphite ligands (**2a**, **2b**, **3b** and **3c**).

Iron(II) complexes	Fully paired DNA (kcal/mol)	Mismatched DNA (kcal/mol)
2a	− 4.35	− 12.80
2b	− 3.76	− 11.74
3b	− 4.86	− 11.86
3c	− 1.84	− 9.24

### Results of the DNA titration study

**Figure 7:**
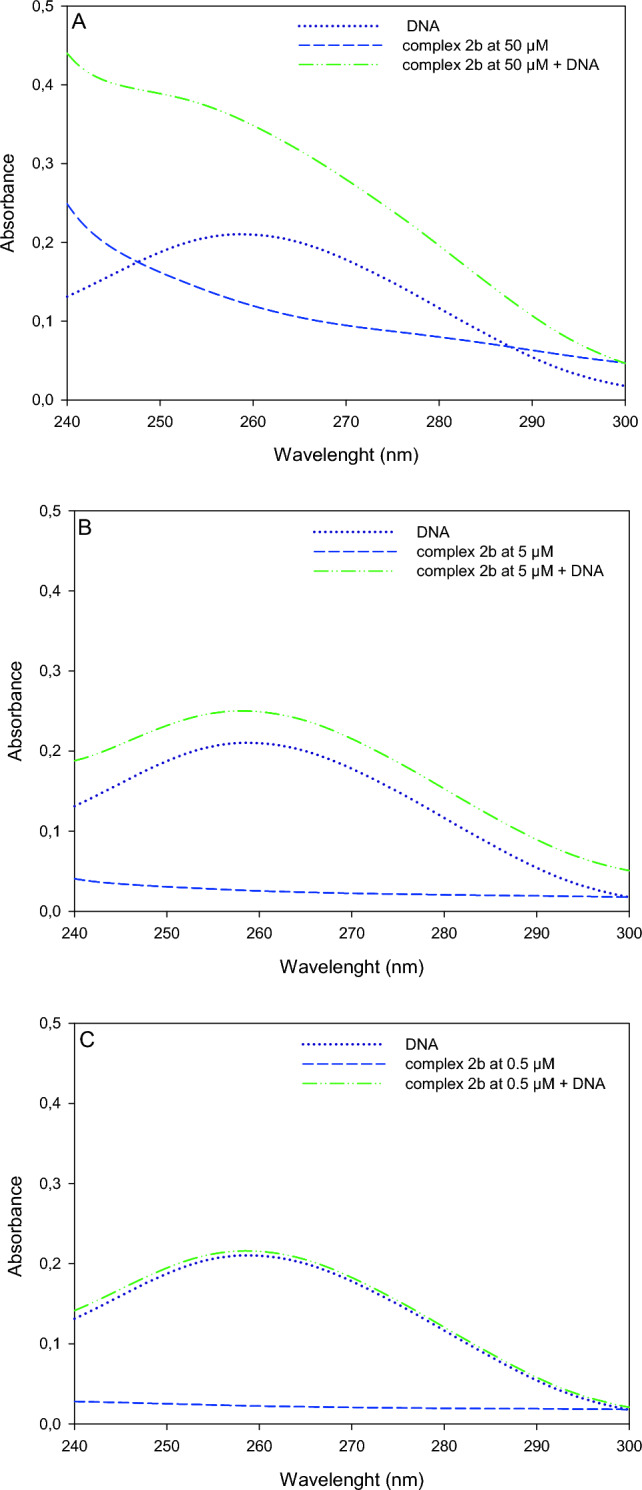
UV–Vis absorption spectra (240–300 nm) of DNA and complex **2b** at 50 µM (**A**), 5 µM (**B**), and 0.5 µM (**C**).

We performed UV–Vis spectroscopic analysis to assess whether these iron(II) complexes can impose structural changes in DNA structure. For this purpose, we analyzed UV–Vis absorption spectra (240–300 nm) for DNA, complex **2b**, and DNA incubated with complex **2b** at 0.5, 5, and 50 µM (Fig. 7). We deliberately chose complex **2b** for this study as the most potent agent with anticancer properties.

In this study, we analyzed UV–Vis absorption spectra (240–300 nm) of DNA, complex **2b** alone and after incubation with DNA. First of all, a significant absorbance for complex **2b** at 260 nm was observed restricting the analysis (Fig. 7A). We observed an increase in absorbance at 260 nm when DNA was incubated with complex **2b** (Fig. 7), however, we attribute this change to the additive effect of absorbance for complex **2b** and DNA, measured separately. Since we have not observed hyperchromic, hypochromic, hypsochromic or bathochromic effect we exclude the possibility of inducing DNA structural changes by complex formation with **2b**. The spectroscopic titration is a sensitive method for assessing changes in DNA structure especially by intercalators, which insert a planar aromatic ring between the stacked base pairs of double-stranded DNA^38^. Thus, we can conclude that complex **2b** does not interact in an intercalative manner with DNA.

## Conclusions

We designed and investigated the potential anticancer activity of five iron(II) cyclopentadienyl complexes bearing different phosphine and phosphite ligands. All complexes have been characterized by ^1^H NMR, ^13^C NMR, ^31^P NMR, FT–IR, ESI–MS, UV–Vis, elemental analyses and single crystal X-ray diffraction analysis (for **2a**, **2b** and **3b**, **3c**). Our biological studies indicated that the complex **2b** bearing tri(2-furyl)phosphine was cytotoxic for both cancer cell lines, HL-60 and A549 cells and not for the normal PBM cells. Having considered the results of the present work with our previous findings regarding Ru complexes^16,39^ it can be postulated that both ruthenium and iron-based (η^5^-cyclopentadienyl) piano stool complexes bearing maleimide and carbonyl ligands exert anticancer activity against HL-60 cells. It is worth noting that complex **3a** and its ruthenium analogue **3a-Ru** were potent against HL-60 cells without cytotoxicity toward PBM cells, indicating that the triethyl phosphite ligand can effect selectivity toward HL-60 cancer cells^16^.

The replacement of the central atom in both complexes containing phosphines, **2a** and **2b**, modulated the cytotoxicity toward PBM cells and HL-60 cells. On one hand, iron-bearing complex **2b** displayed no cytotoxicity toward PBM cells compared to its ruthenium counterpart **2b-Ru** which is cytotoxic. On the other hand, the complex **2a** showed cytotoxicity toward PBM cells compared to its non-toxic ruthenium analogue **2a-Ru**^16^. This observation led us to the conclusion that the substitution of central atom in metal containing complexes can influence their selectivity which is crucial for bioinorganic chemical activity.

Interestingly, comparing the activity of complex **1** to complex **3c** bearing triisopropyl phosphite we did not observe differences in the cytotoxicity toward HL-60 cells and PBM cells, but in case of A549 cells the **3c** was no cytotoxic when **1** showed significant cytotoxicity.

The DFT studies showed that the complexes **2a** and **3c** should be the most active; however, we demonstrated their high cytotoxic activity mainly towards HL-60 leukemic cells. These complexes were not cytotoxic to A549 cells (IC_50_ > 250 µM). We also found that the complex **2b** induced DNA damage in both types of cancer cells. It is noteworthy that all the studied compounds were less toxic than complex **1** that is lacking phosphine and phosphite ligand. Molecular docking revealed that complexes **2a**, **2b** and **3b**, **3c** have potential to bind to mismatched DNA. However, the plasmid relaxation assay showed that these iron(II) complexes did not induce the DNA breaks. Furthermore, the DNA titration experiment showed no intercalation of complex **2b** into DNA. Our studies demonstrated that the iron(II) complex **1** and **2b** are the most interesting compounds in terms of selective cytotoxic action against cancer cells. Therefore, we suggest that DNA damage probably occurs as a result of impaired cellular metabolic processes under the influence of iron(II) complexes rather than direct interaction with DNA.

## Materials and methods

### Chemicals

Bis(cyclopentadienylirondicarbonyl) dimer, maleimide, diisopropylamine, and all the phosphines and phosphites were purchased from Sigma-Aldrich (Merck). Solvents were purchased from POCH (Polish Chemical Reagents) and used without further purification. All syntheses were carried out under argon. Chromatographic purification of the crude products were performed on silica gel 60 (230–400 mesh) purchased from Merck. FTIR spectra were recorded in KBr on a Fourier Transform InfraRed (FTIR) NEXUS (Thermo Nicolet) spectrometer. NMR spectra were recorded on Bruker Avance III BBFO (600 MHz) and Bruker AvanceNeo Cryoprobe Prodigy spectrometer (600 MHz). NMR data were collected in CDCl_3_ (Merck) solution. The chemical shifts were calculated in part per million (ppm) unit. Coupling constants were calculated in Hertz (Hz). Electrospray ionization mass spectrometry (ESI–MS) spectra were recorded on the Varian 500-MS LC ion trap spectrometer. Elemental analyses were obtained with a Vario EL III (Elementar Analysensysteme GmbH) instrument. Photochemical syntheses were carried out using UV lampTQ 150 Z3. PerkinElmer Lambda 45 UV/Vis spectrometer and PerkinElmer LS55 Fluorimeter have been used to measure the absorbance and emission of all compounds, respectively. Relevant guidelines and regulations were followed in each consecutive step. Dulbecco's Modified Eagle Medium (DMEM), IMDM medium and fetal bovine serum (FBS) were obtained from Biowest (Cytogen, Zgierz, Poland). Dimethyl sulfoxide (DMSO), hydrogen peroxide (H_2_O_2_), low-melting-point (LMP), normal-melting-point (NMP) agarose, phosphate buffered saline (PBS), and 4ʹ,6-diamidino-2-phenylindole (DAPI) were purchased from Sigma-Aldrich (USA). The pUC19 plasmid isolation kit (Plazmid Mini AX Kit) was obtained from A&A Biotechnology and restrictase *Pst*I from New English Biolabs. All other reagents were obtained at the highest commercially available grades. A stock solutions of iron complexes (10 mM) was dissolved in DMSO.

### Synthetic procedures

Complex **1** was synthesized according to previously published method, by photochemical reaction of CpFe(CO)_2_I with maleimide in the presence of diisopropyl amine in toluene^24^.

### General procedure for synthesis of 2a,b and 3a–c

A stirred, water–ice cooled, and argon-saturated solution of **1** (70 mg, 0.25 mmol) and phosphine or phosphite (0.7 equiv.) was illuminated under visible light (4 × 150W lamps) for 2 h in toluene (10 mL). The progress of the reaction was continuously monitored with TLC. After completion of the reaction, solvent was evaporated in vacuum.

**Synthesis of 2a** The crude product was purified by column chromatography using CHCl_3_-EtOAc (3:1) as eluent to afford a brown–red solid (**2a**). The product was recrystallized from chloroform/heptane. Yield 32 mg (55%). ^1^H NMR (δ, ppm, 600 MHz, CDCl_3_): 7.38 (dd, J = 7.2 Hz, 6.6 Hz, 3H, *p*-Ph), 7.32 (m, 12H, *o,m*-Ph), 6.17 (s, 2H, olefinic), 4.60 (s, 5H, Cp). ^13^C NMR (δ, ppm, 151 MHz, CDCl_3_): 221.79 (d, J = 30.6 Hz, C≡O); 185.23 (s, imide C=O); 137.41 (s, olefinic C=C); 133.98 (d, J = 42.3 Hz, phenyl C–P); 133.69 (d, J = 10 Hz, *o*-C of Ph); 130.07 (d, J = 1.8 Hz, *p*–C of Ph); 128.35 (d, J = 9.7 Hz, *m*-C of Ph); 82.65 (s, Cp). ^31^P NMR (δ, ppm, 242.99 MHz, CDCl_3_): 75.27. FTIR (cm^−1^): 1951 (C≡O); 1639 (C=O, imide); 1327, 695 (P-Ph). ESI–MS: m/z calcd for C_28_H_22_FeNO_3_P (M + H)^+^, 508.07; found, 508.13. Anal. calcd for C_28_H_22_FeNO_3_P (507.0687): C 66.29; H 4.37; N 2.76; found C 66.17; H 4.35; N 2.57.

**Synthesis of 2b** The crude product was purified by column chromatography using CHCl_3_-EtOAc (3:2) as eluent to afford a red solid (**2b**). The product was recrystallized from chloroform/heptane. Yield: 56 mg (68%). ^1^H NMR (δ, ppm, 600 MHz, CDCl_3_): 7.64 (s, 3H, furyl H), 6.71 (d, J = 3.6 Hz, 3H, furyl H), 6.44 (ddd, J = 1.2 Hz, 3 Hz, 3H, furyl H), 6.41 (s, 2H, olefinic), 4.80 (d, J = 1.2 Hz, 5H, Cp). ^13^C NMR (δ, ppm, 151 MHz, CDCl_3_): 219.25 (d, J = 31.6 Hz, C≡O); 185.13 (s, imide C=O); 147.89 (d, J = 5.2 Hz, furyl C–O); 146.86 (d, J = 70.8 Hz, furyl C–P); 137.67 (s, olefinic C=C); 121.44 (d, J = 15.7 Hz, furyl C=C); 111.35 (d, J = 6.7 Hz, furyl C=C); 82.48 (s, Cp). ^31^P NMR (δ, ppm, 242.99 MHz, CDCl_3_): 29.25. FTIR (cm^−1^): 1966 (C≡O); 1645 (C=O, imide); 1019, 758 (P-Fu). ESI–MS: m/z calcd. for C_22_H_16_FeNO_6_P (M + H)^+^, 478.0098; found, 478.17. Anal. calcd for C_22_H_16_FeNO_6_P (477.0065): C 55.37; H 3.38; N 2.94; found C 55.33; H 3.15; N 2.75.

**Synthesis of 3a** The crude product was purified by column chromatography using EtOAc–Petroleum Ether (1:1) as eluent to afford a brown–red liquid product (**3a**). The product was recrystallized from chloroform/heptane. Yield 38 mg (79%). ^1^H NMR (δ, ppm, 600 MHz, CDCl_3_): 6.53 (s, 2H, olefinic), 4.70 (s, 5H, Cp), 3.92–3.86 (m, 6H, –CH_2_), 1.22 (t, J = 7.06 Hz, 9H, –CH_3_). ^13^C NMR (δ, ppm, 151 Hz, CDCl_3_): 220.11 (d, J = 45 Hz, C≡O); 185.33 (s, imide C=O); 137.86 (s, olefinic C=C); 82.57 (s, Cp); 61.47 (s, -CH_2_); 16.32 (s, –CH_3_). ^31^P NMR (δ, ppm, 242.99 MHz, CDCl_3_): 175.37. FTIR (cm^−1^): 1972 (C≡O); 1649 (C=O, imide); 1032, 947 (P-OEt). ESI–MS: m/z calcd. for C_12_H_22_FeNO_6_P (M + H)^+^, 412.05; found, 412.11. Anal. calcd for C_16_H_22_FeNO_6_P (411.0534): C 46.74; H 5.39; N 3.41; found C 46.53; H 5.34; N 3.24.

**Synthesis of 3b** The crude product was purified by column chromatography using CHCl_3_-EtOAc (3:1) as eluent to afford an orange solid (**3b**). The product was recrystallized from chloroform/heptane. Yield 50 mg (70%). ^1^H NMR (δ, ppm, 600 MHz, CDCl_3_): 7.31–7.28 (m, 6H, *meta*-H of phenyl), 7.14 (dd, J = 7.9 Hz, 13.8 Hz, 9H, *ortho*-, *para*-H of phenyl), 6.35 (s, 2H, olefinic), 4.60 (s, 5H, Cp). ^13^C NMR (δ, ppm, 151 Hz, CDCl_3_): 218.30 (d, J = 42 Hz, C≡O); 184.72 (s, imide C=O); 151.33 (d, J = 9 Hz, phenyl C–O); 137.60 (s, olefinic C=C); 129.78 (s, *meta*-C of Ph); 125.14 (s, *ortho*-C of phenyl); 121.26 (d, J = 4.5 Hz, *para*-C of phenyl); 82.85 (s, Cp). ^31^P NMR (δ, ppm, 242.99 MHz, CDCl_3_): 168.28. FTIR (cm^−1^): 1970 (C≡O); 1646 (C=O, imide); 1194, 921 (P-OPh). ESI–MS: m/z calcd for C_28_H_22_FeNO_6_P (M + H)^+^, 556.05; found, 556.21. Anal. calcd for C_28_H_22_FeNO_6_P (555.0534): C 60.56; H 3.99; N 2.52, found C 60.66; H 3.96; N 2.4.

**Synthesis of 3c** The crude product was purified by column chromatography using CHCl_3_-EtOAc (3:1) as eluent to afford brown–red crystals (**3c**). The product was recrystallized from chloroform/heptane. Yield 38 mg (38%). ^1^H NMR (δ, ppm, 600 MHz, CDCl_3_): 6.54 (s, 2H, olefinic), 4.65 (s, 5H, Cp), 4.45–4.39 (m, 3H of ^*i*^Pr), 1.22 (dd, J = 9 Hz, 6 Hz, 18H of ^*i*^Pr). ^13^C NMR (δ, ppm, 151 Hz, CDCl_3_): 220.45 (d, J = 48 Hz, C≡O); 185.47 (s, imide C=O); 137.92 (s, olefinic C=C); 82.87 (s, Cp); 69.85 (d, J = 6 Hz, CH of ^*i*^Pr); 24.20 (t, J = 4.5 Hz, CH_3_ of ^*i*^Pr). ^31^P NMR (δ, ppm, 242.99 MHz, CDCl_3_): 170.93. FTIR (cm^−1^): 1964 (C≡O); 1647 (C=O, imide); 1168, 1006, 696 (P-O*i*Pr). ESI–MS: m/z calcd. for C_19_H_28_FeNO_6_P (M + H)^+^, 454.10; found, 454.36. Anal. calcd for C_19_H_28_FeNO_6_P (453.1004): C 50.35; H 6.23; N 3.09; found C 50.32; H 6.04; N 2.96.

### X-ray structure determination

X-ray diffraction data for **2a, 2b, 3b** and **3c** compounds were measured on a four-circle Oxford Diffraction Supernova Dual diffractometer using a two-dimensional area CCD detector and a low-temperature device Oxford Cryosystem cooler. Integration of the intensities, corrections for Lorentz effects, polarization effects and analytical absorption were performed with CrysAlis PRO^40^. The crystal structures were solved by direct methods and refined on *F*^2^ using a full-matrix least-squares procedure (SHELXL-2014)^41^. During anisotropic refinement there were observed some evidence of crystal disorder (elongated displacement ellipsoids) in case of **3b**. Finally the crystal structure was refined as a disordered one with two positions of cyclopentadienyl ring of occupancies ratio 0.56:0.44. Moreover, in the refinement additional restrains were applied to displacement parameters of carbon atoms (C11A/B, C12A/B, C13A/B, C14A/B, C15A/B) including SADI instruction in Shelx.

In all the investigated crystal structures the positions of the hydrogen were introduced in the calculated positions with an idealized geometry and constrained using a rigid body model with isotropic displacement parameters equal to 1.2 of equivalent displacement parameters of their parent atoms. The molecular geometry was calculated by Platon^42^ and WinGX programs^43^. The relevant crystallographic data are given in Table 2. Atomic coordinates, displacement parameters, an structure factors of the analysed crystal structures are deposited with Cambridge Crystallographic Data Centre CCDC^44^. Deposit numbers are submitted in the Table 2.

### Cell culture

The A549 (human non-small cell lung cancer) cell line was obtained from the American Type Culture Collection (ATCC) and cultured in Dulbecco's Modified Eagle Medium (DMEM) with 10% fetal bovine serum (FBS), 2 mM l-Glutamine, 25 mM HEPES and penicillin/streptomycin solution (100 U/ml and 100 µg/ml, respectively). The HL-60 (human promyelocytic leukemia) cell line was also obtained from the American Type Culture Collection (ATCC) and cultured in Iscove’s Modified Dulbecco's Medium (IMDM) with 15% fetal bovine serum (FBS), 2 mM l-Glutamine, 25 mM HEPES and penicillin/streptomycin solution (100 U/ml and 100 µg/ml, respectively). Both cell lines were cultured in flasks at 37 °C in 5% CO_2_ and sub-cultured every 2–3 days to maintain exponential growth.

Peripheral blood mononuclear (PBM) cells were isolated from a leukocyte-buffy coat collected from the blood of healthy, non-smoking donors at the Blood Bank in Lodz, Poland. The study protocol received approval from the Committee for Research on Human Subjects of the University of Lodz (17/KBBN-UŁ/III/2019). The initial step of isolation process involved mixing buffy coats with PBS in a 1:1 ratio. Subsequently, the mixture was centrifuged using a density gradient of Lymphosep (Cytogen, Zgierz, Poland) at 2200 RPM for 20 min, employing the lowest values for acceleration and deceleration. PBM cells were collected and washed three times by centrifugation with 1 × PBS. Following isolation, the cells were suspended in RPMI 1640 medium.

### Cell viability resazurin assay

Firstly, resazurin salt powder was dissolved in sterile 1 × PBS. Cells were then seeded on the 96-well plates in the count of 1 × 10^4^ in the case of HL-60 cells and of 5 × 10^4^ for PBM cells and of 5 × 10^3^ for A549 cells per well. A549 cells were seeded 24 h prior to treatment with the complexes to allow cell adherence to 96-well plate. Iron(II) complexes were added to wells to obtain final concentrations of 0.5, 1, 2.5, 5, 10, 25, 50, 100 and 250 μM. Subsequently, plates were incubated at 37 °C in 5% CO_2_ for 2 h and 24 h. After that 10 μl of resazurin salt was added to each well and plates again were incubated in 37 °C in 5% CO_2_ for 2 h. Finally, fluorescence was measured with microplate reader Synergy HT (Bio-Tek Instruments, USA) using an excitation wavelength of 530/25 and an emission wavelength of 590/35 nm. The effects of iron(II) complexes were calculated as the percentage of control fluorescence. IC_50_ (half maximal inhibitory concentration) values were calculated using a website algorithm (https://www.aatbio.com/tools/ic50-calculator). All assays were performed in octuplicate.

### DNA damage by the comet assay

Selected iron(II) complexes were added to the suspension of the cells to give final concentrations of 1, 5, 10, 25 and 50 μM. HL-60 and A549 cells were incubated with the complexes for 2 h at 37 °C. The comet assay was performed under alkaline conditions according to the procedure of Tokarz et al.^45^. After incubation, a freshly prepared cells suspension in 0.75% LMP agarose dissolved in PBS was layered onto microscope slides (Superior, Germany), which were pre-coated with 0.5% NMP agarose. Next, the cells were lysed for 1 h at 4 °C in a buffer containing 2.5 M NaCl, 0.1 M EDTA, 10 mM Tris, 1% Triton X-100, pH 10. Following the lysis, the slides were placed in an electrophoresis unit. DNA was allowed to unwind for 20 min in the solution containing 300 mM NaOH and 1 mM EDTA, pH > 13. Electrophoretic separation was performed in the solution containing 30 mM NaOH and 1 mM EDTA, pH > 13 at ambient temperature of 4 °C (the temperature of the running buffer did not exceed 12 °C) for 20 min at an electric field strength of 0.73 V/cm (28 mA). After separation, the slides were washed in water, drained, stained with 2 μg/ml DAPI and covered with cover slips. As a prevention from additional DNA damage, the procedure described above was conducted under limited light or in the dark. Each experiment included the positive control—cells incubated with H_2_O_2_ at 20 µM for 15 min on ice (data not shown).

### Comet analysis

The comets obtained after single cell gel electrophoresis were analysed in the following manner. The comets were observed at 200× magnification in an Eclipse fluorescence microscope (Nikon, Japan) attached to a ProgRes MF cool monochrome video camera (JENOPTIK, Jena, Germany) and connected to a personal computer-based image analysis system Lucia Comet Assay version 7.30 (Laboratory Imaging, Praha, Czech Republic). Hundred images of singular comets were randomly selected from each sample and the mean value of DNA in comet tail was taken as an index of DNA damage (expressed in percent).

### Plasmid relaxation assay

The pUC19 plasmid was isolated from the DH5α *E. coli* cells with Plasmid Mini AX Kit (A&A Biotechnology) according to the manufacturer’s instruction. The isolated plasmid quantity and quality were determined by A260/A280 ratio and gel electrophoresis, respectively. The native form of pUC19 exists mainly in the supercoiled form (CCC) which is characterized by a relatively high electrophoretic mobility. The plasmid was digested with the restrictase *Pst*I (New English Biolabs) to induce linear (L) form. Topological differences between CCC and L forms of the plasmid account for their different electrophoretic mobility. The plasmid at 50 ng/μl was incubated for 2 h and 24 h with iron(II) complexes at the concentration of 5 µM and 50 µM. Then the samples were subjected to 1% agarose gel electrophoresis with ethidium bromide staining, visualization under UV light (302 nm), scanning by a CCD camera, and analysis with the GeneTools by Syngene (Cambridge, UK) software. During electrophoresis, we also separated 4 μl of 1 kb DNA ladder (GeneRuler 1 kb DNA Ladder, Thermo Scientific, Waltham, MA, USA).

### Docking studies

The molecular docking was performed using AutoDock^46^ with the grid box designed based on the DNA structure as a substrate with iron(II) complex as a ligand. Fully paired 12nt DNA fragment was used as an example of ds-DNA. For the mismatched DNA also 12nt fragment with T-T mismatch was selected and structure optimized as in previous study^16^ to mimic damaged DNA strand. To produce global structure of complexes, a grid-based docking was applied using the Lamarckian genetic algorithm. Appropriate parameters for iron(II) were added to the AutoDock parameters file. Every docking was analysed as 10 best possible conformations with 3 repeats. Metal complex ligand conformations of the lowest energy, was analysed and if necessary used for additional MD simulations.

### DNA titration

Genomic DNA was isolated from A549 cell line using a commercially available kit (Extractme Genomic DNA Kit, Blirt, Gdansk, Poland) following the manufacturer’s protocol. The concentration and purity of DNA were measured by BioTek Synergy HT Microplate Reader (BioTek Instruments, Winooski, VT, USA). Genomic DNA in the amount of 20 µg was incubated with complex **2b** for 15 min at room temp. at concentrations 0.5, 5, and 50 µM in a final volume of 2 ml. Next UV–Vis absorbance spectra (240–300 nm) was measured by UV–Vis JASCO V-630 spectrophotometer (JASCO, Japan).

### Supplementary Information

Supplementary Information.

## Data Availability

All data generated or analysed during this study are included in this published article and its supplementary information files.
