# Supplementary material for: Synthesis, anticancer activity, and molecular docking of half-sandwich iron(II) cyclopentadienyl complexes with maleimide and phosphine or phosphite ligands
Source: Sci Rep. 2024 Mar 7;14:5634. doi: 10.1038/s41598-024-56339-0 (PMC10920834; doi:10.1038/s41598-024-56339-0)
Supplement: Supplementary file 1 — Supplementary Information. [file 41598_2024_56339_MOESM1_ESM.pdf]

Synthesis, anticancer activity, and molecular docking of half-sandwich Iron(II) cyclopentadienyl complexes with maleimide and phosphine or phosphite ligands

Sujoy Das,[a] Marcelina Strachanowska,[b] Piotr Wadowski,[b] Michał Juszczak,[b] Paulina Tokarz,[b] Aneta Kosińska,[a] Marcin Palusiak,[c] Agnieszka J. Rybarczyk-Pirek,[c] Kinga Wzgarda-Raj,[c] Saranya Vasudevan,[d] Arkadiusz Chworos,[d] Katarzyna Woźniak, *[b] Bogna Rudolf *[a]

[a] University of Lodz, Faculty of Chemistry, Department of Organic Chemistry, Tamka 12, 91-403 Lodz, Poland.

[b] University of Lodz, Faculty of Biology and Environmental Protection, Department of Molecular Genetics, Pomorska 141/143, 90-236, Lodz, Poland.

[c] University of Lodz, Faculty of Chemistry, Department of Physical Chemistry, Pomorska 163/165, 90-236 Lodz, Poland.

[d] Centre of Molecular and Macromolecular Studies, Polish Academy of Sciences, Sienkiewicza 112, 90-363 Lodz, Poland.

Address for correspondence: Bogna Rudolf, e-mail: bogna.rudolf@chemia.uni.lodz.pl, Katarzyna Woźniak, e-mail: katarzyna.wozniak@biol.uni.lodz.pl.

Supplementary Materials

1. Characterization and spectral analysis

Figure S1-S2. Spectral data of compound **1**

Figure S3-S6. Spectral data of compound **2a**.

Figure S7-S10. Spectral data of compound **2b**.

Figure S11-S14. Spectral data of compound **3a**.

Figure S15-S18. Spectral data of compound **3b**.

Figure S19-S22. Spectral data of compound **3c**.

2. Crystallographic analysis and data

Table S1. Selected bond lengths [Å] and angles [°].

Table S2. Geometric parameters of selected hydrogen bonds–distances [Å] and angles [°].

Figure S23. Schemes of selected hydrogen bonds in the crystal structures.

3. Comparative absorption and emission study

Figure S24. Comparative absorbance spectra of the complexes **2a**, **2b**, **3a**, **3b** and **3c** with respect to corresponding ligands in chloroform.

Figure S25. Comparative emission spectra of the complexes **2a**, **2b**, **3a**, **3b** and **3c** with respect to corresponding ligands in chloroform.

4. Biological studies

Table S3. The viability of PBM, HL-60 and A549 cells after 2 h incubation with iron(II) complexes.

Table S4. The viability of PBM, HL-60 and A549 cells after 24 h incubation with iron(II) complexes.

5. Molecular docking studies

Figure S26. The binding energy values [kcal/mol] obtained by molecular docking of tested compounds **2a-b**, **3b-c** with fully paired ds-DNA and mismatched DNA.

Figure S27. The view of structures for (a) fully paired 12nt ds-DNA fragment will all tested compounds located in minor groove of DNA and (b) mismatched 12nt DNA fragment (T-T mismatch) with all tested compounds located at the level of the mismatch.

1. Characterisation and spectral analyses of the iron(II) complexes:

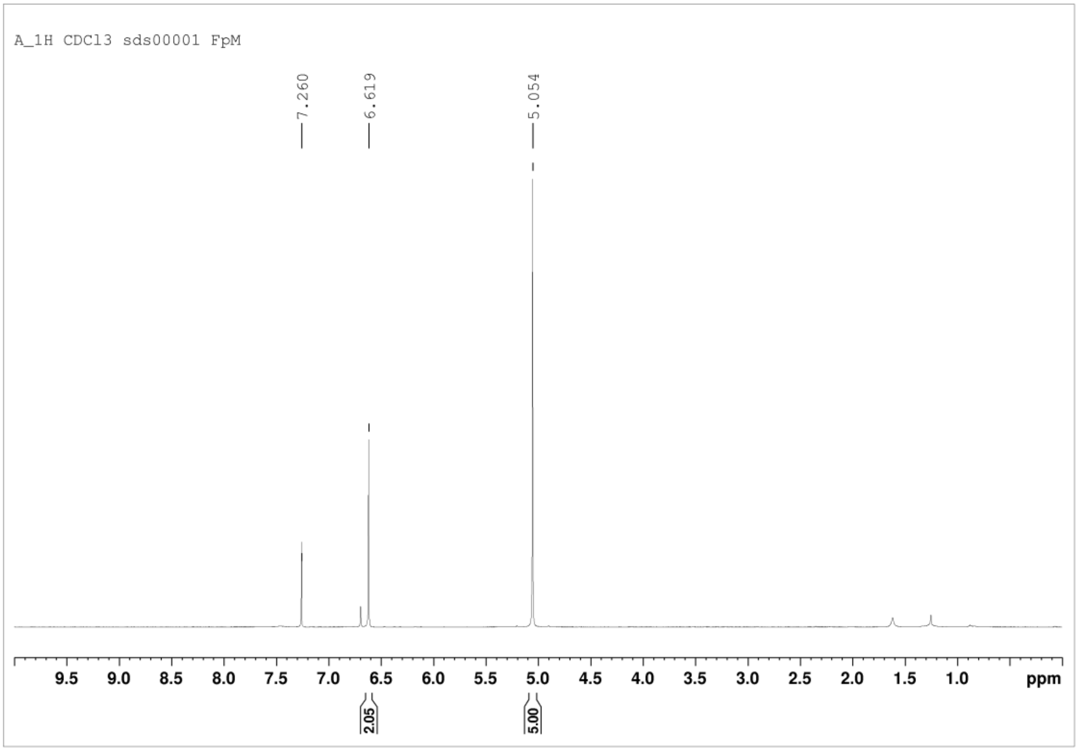

Figure S1. ^{1}H NMR spectra of **1** in $CDCl_3$.

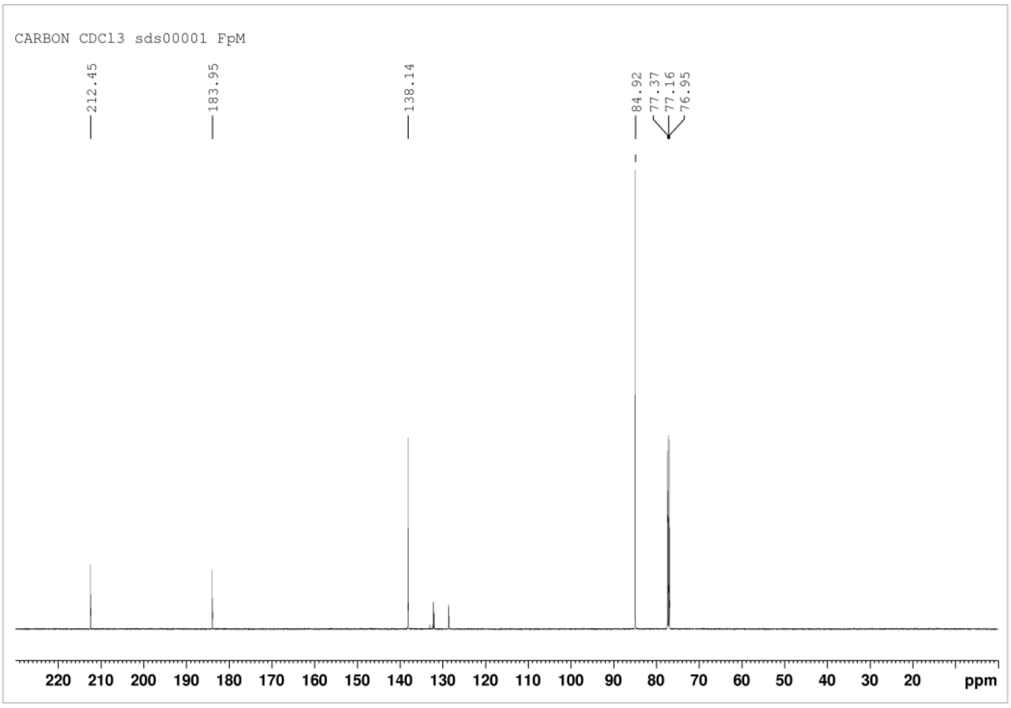

Figure S2. ^{13}C NMR spectra of **1** in $CDCl_3$.

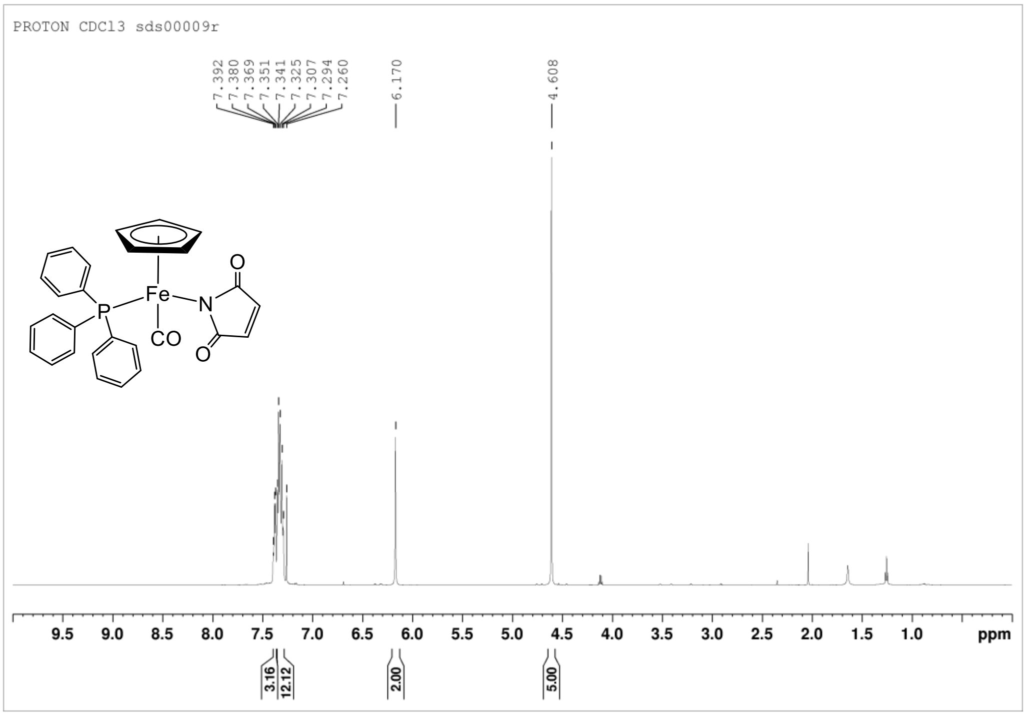

Figure S3. ^{1}H NMR spectra of **2a** in $CDCl_3$.

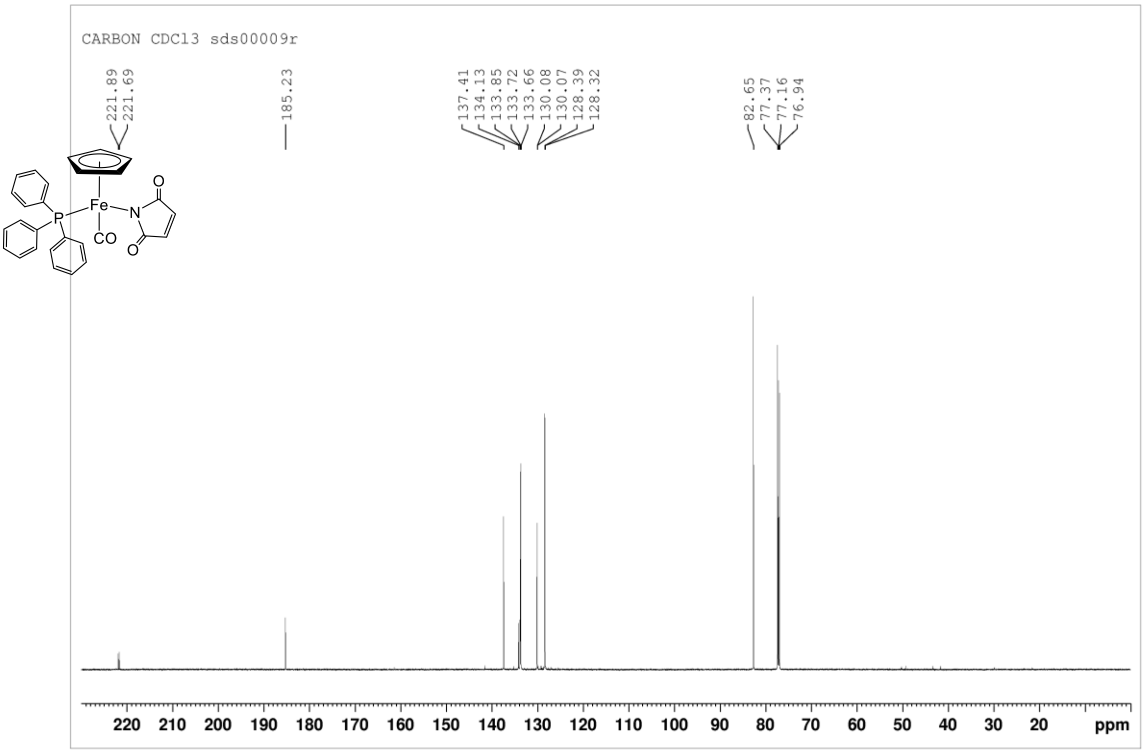

Figure S4. ^{13}C NMR spectra of **2a** in $CDCl_3$.

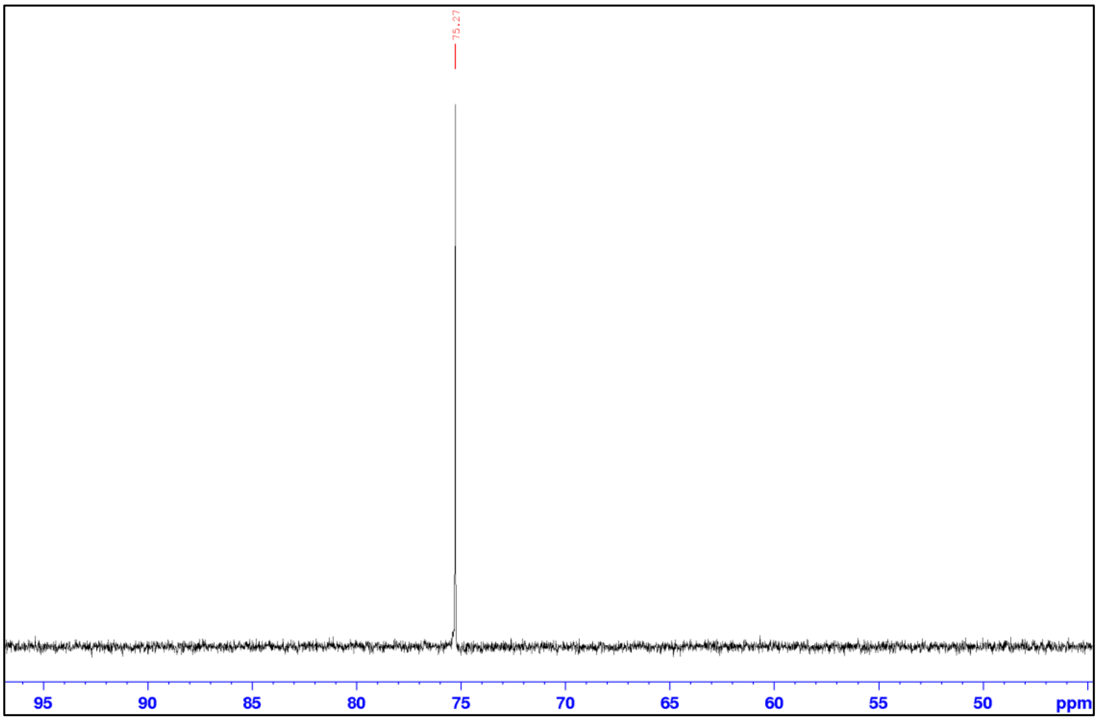

Figure S5. ^{31}P NMR spectra of **2a** in $CDCl_3$.

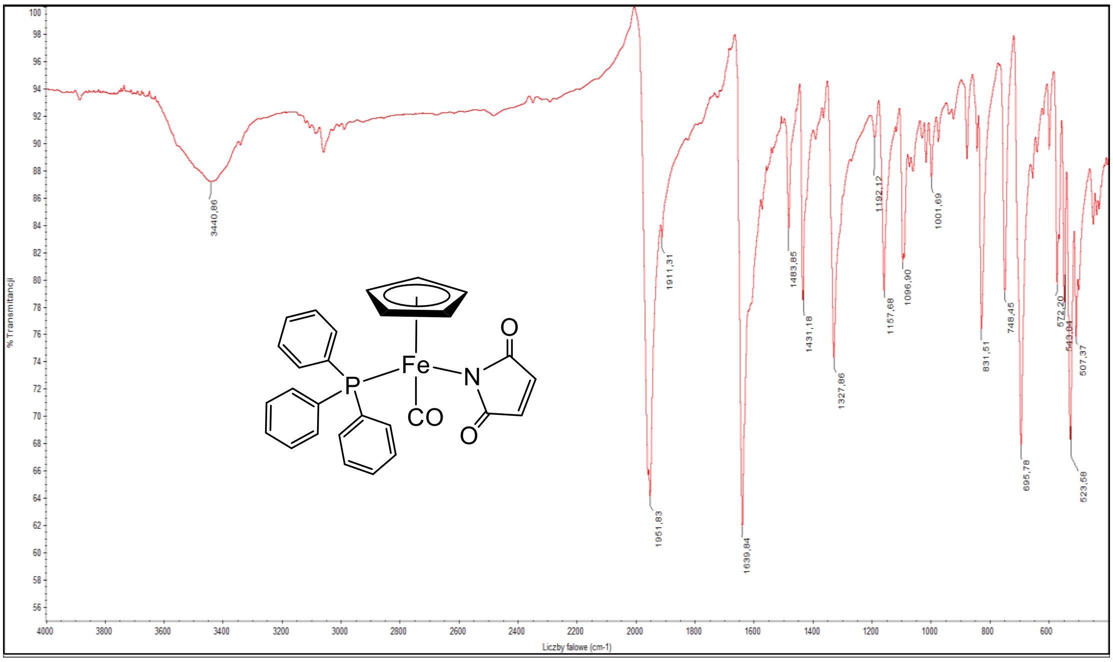

Figure S6. FTIR spectra of **2a**.

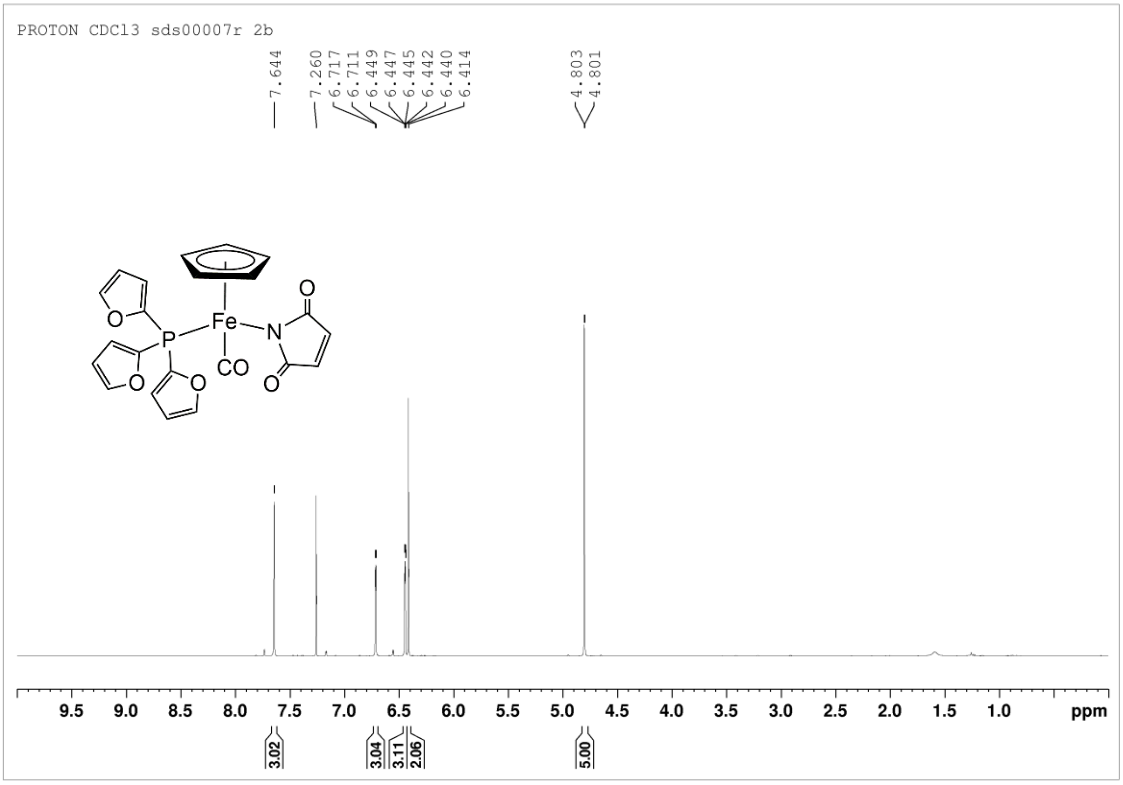

Figure S7. ^{1}H NMR spectra of **2b** in $CDCl_3$.

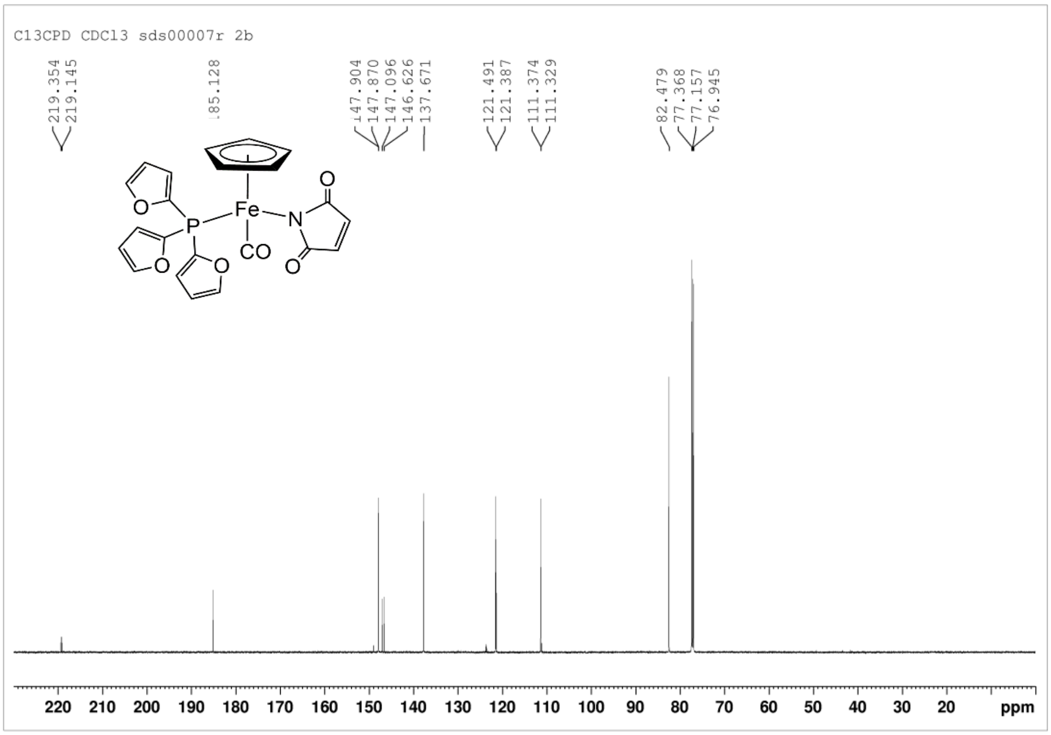

Figure S8. ^{13}C NMR spectra of **2b** in $CDCl_3$.

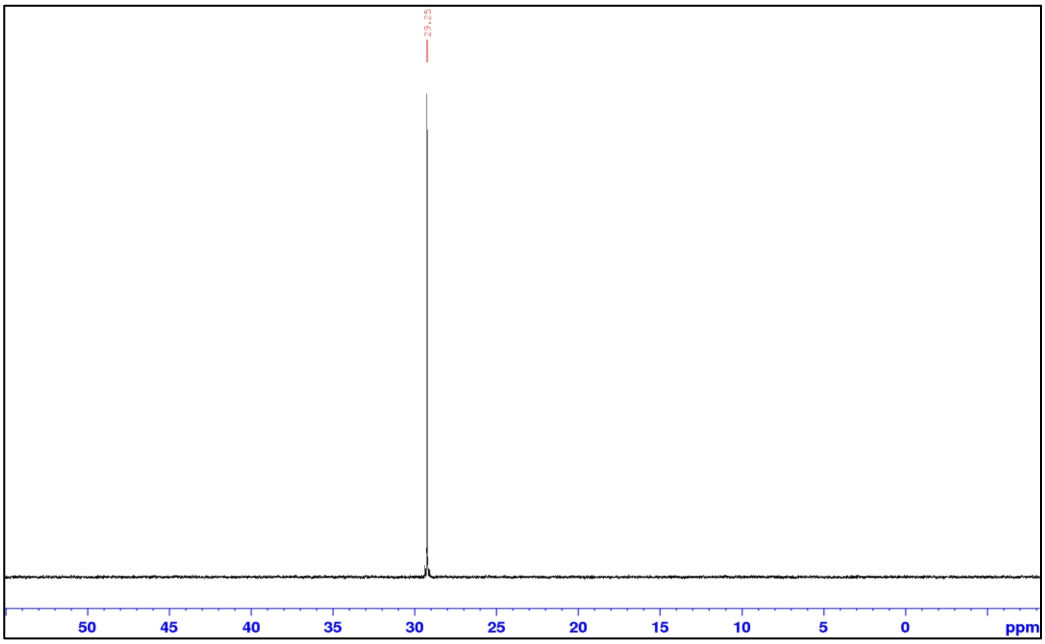

Figure S9. ^{31}P NMR spectra of **2b** in $CDCl_3$.

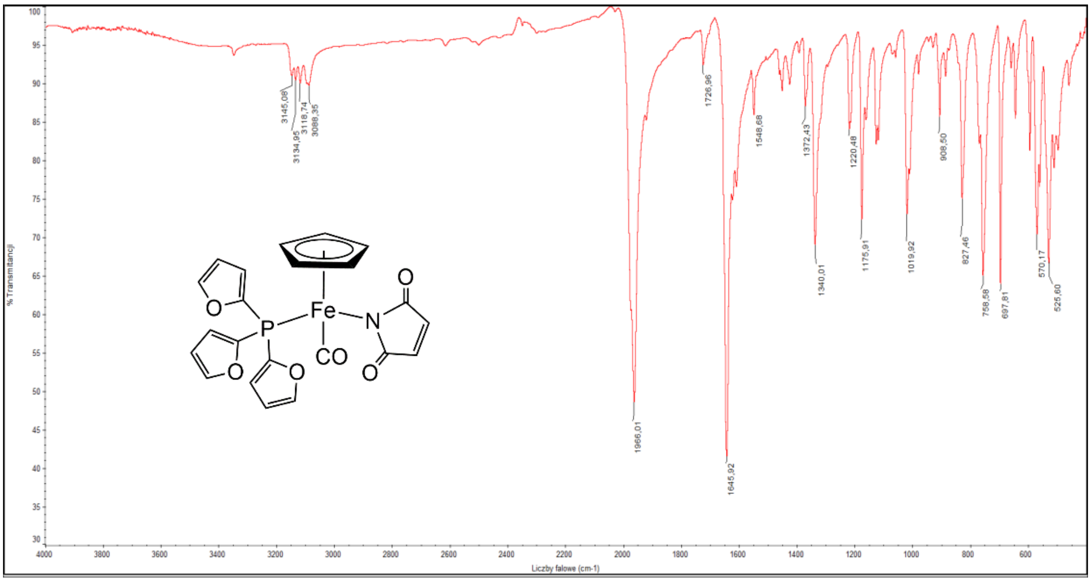

Figure S10. FTIR spectra of **2b**.

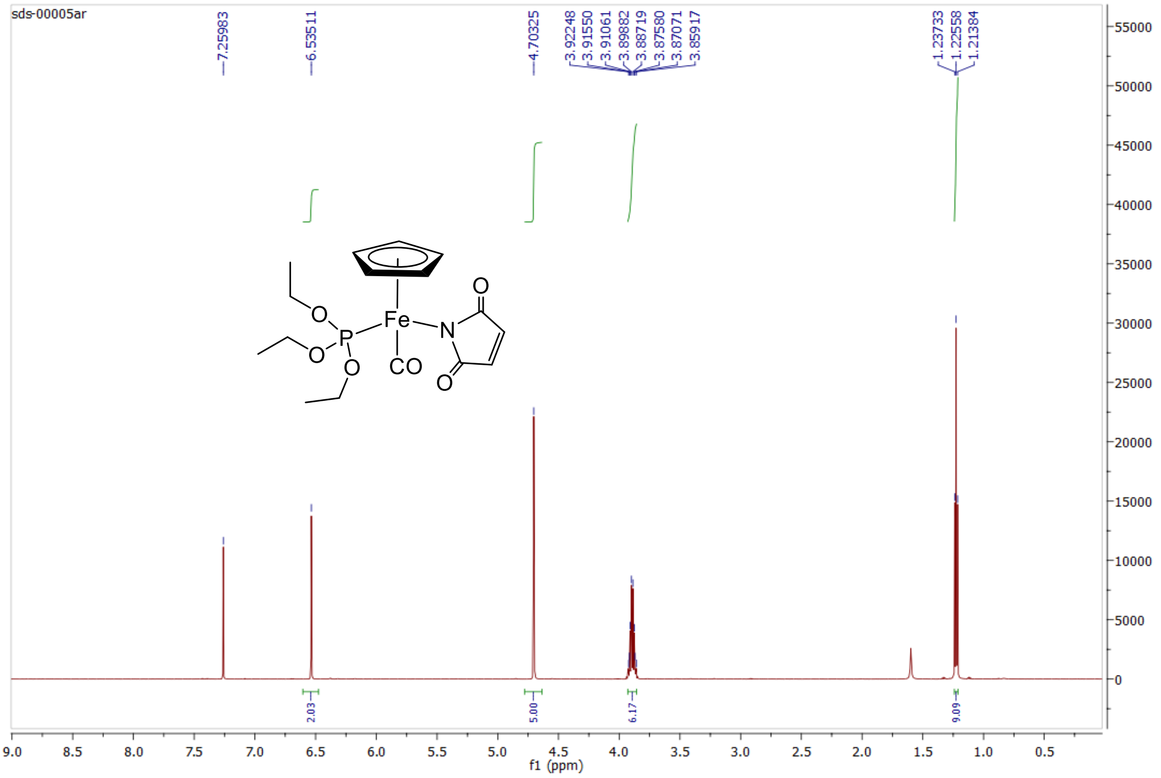

Figure S11. ^{1}H NMR spectra of **3a** in $CDCl_3$.

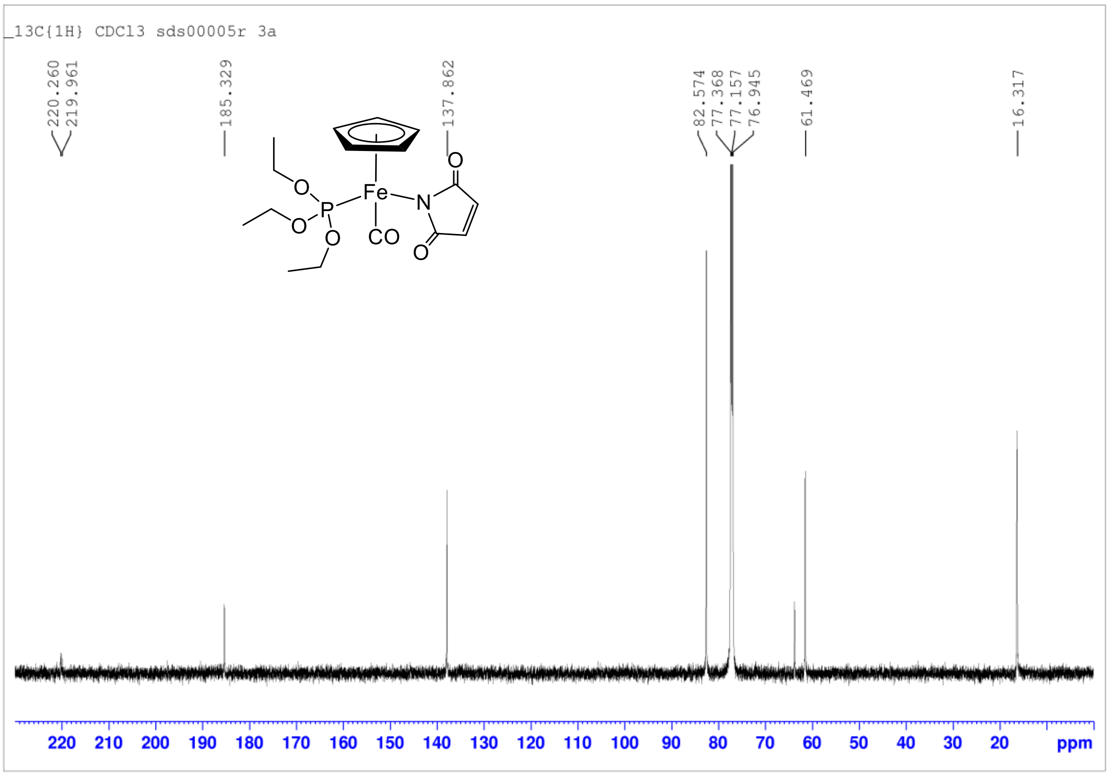

Figure S12. ^{13}C NMR spectra of **3a** in $CDCl_3$.

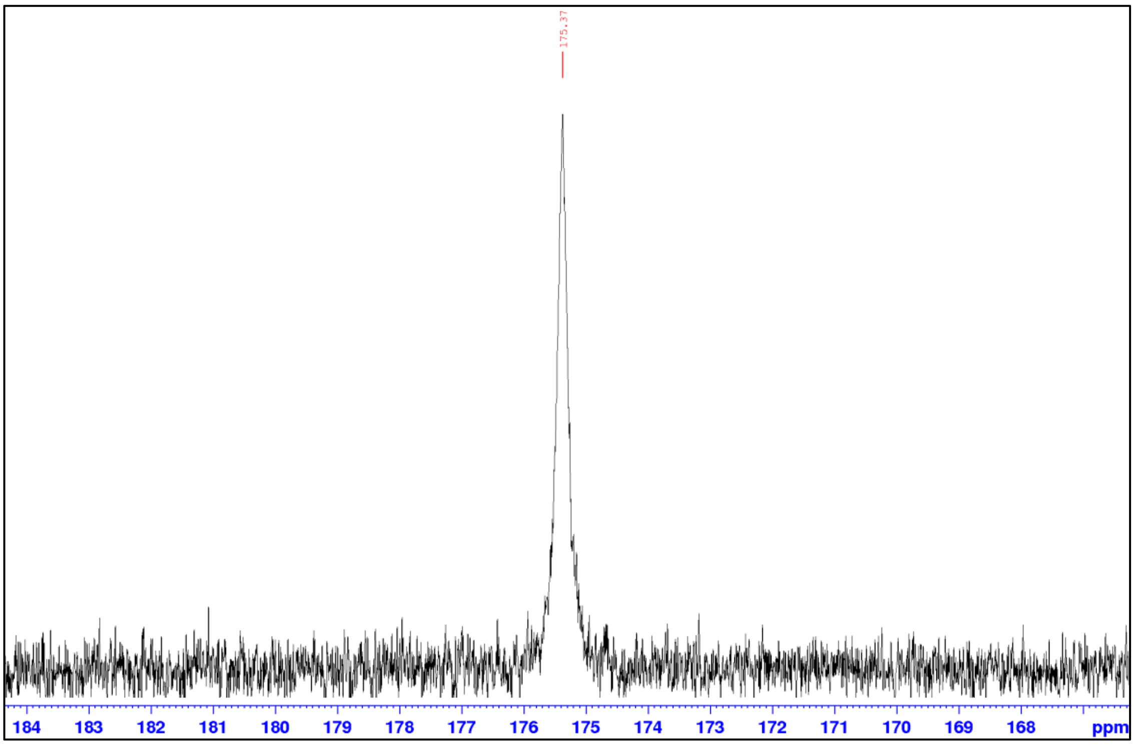

Figure S13. ^{31}P NMR spectra of **3a** in $CDCl_3$.

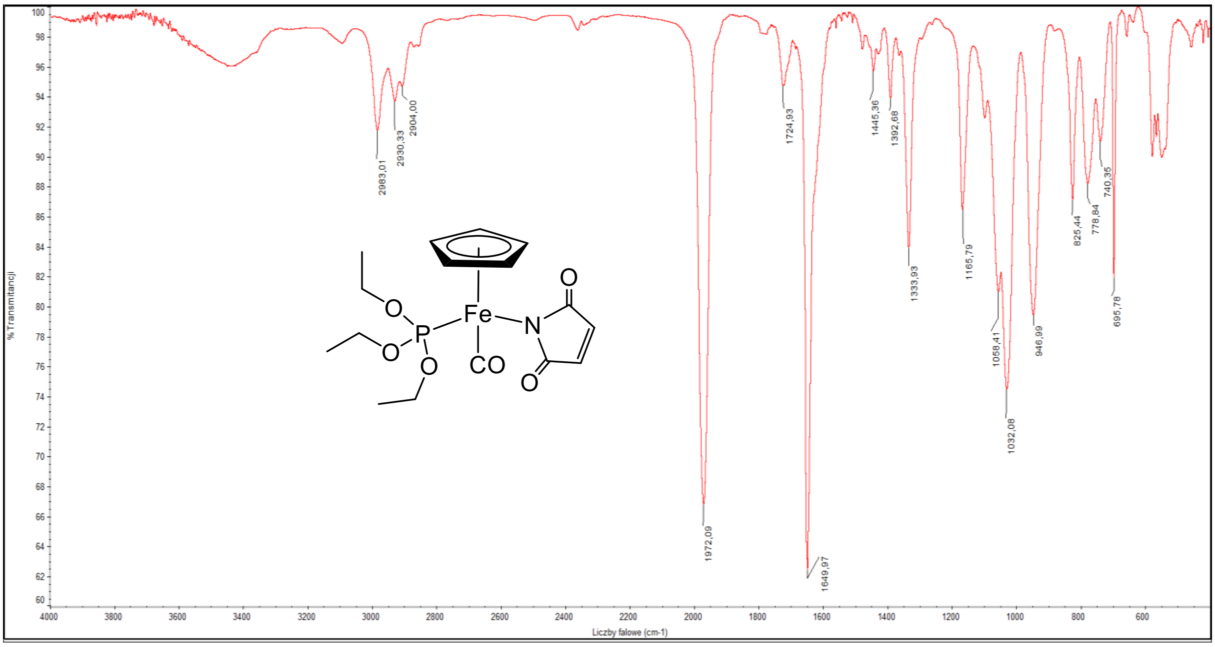

Figure S14. FTIR spectra of **3a**.

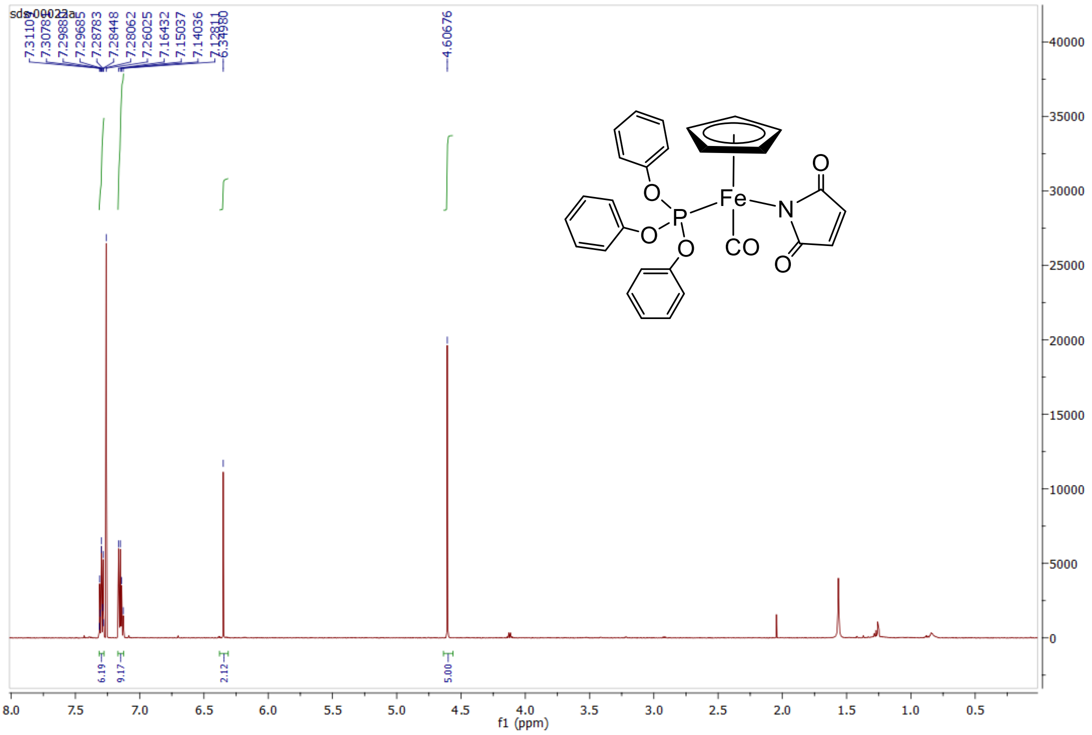

Figure S15. ¹H NMR spectra of **3b** in $CDCl_3$.

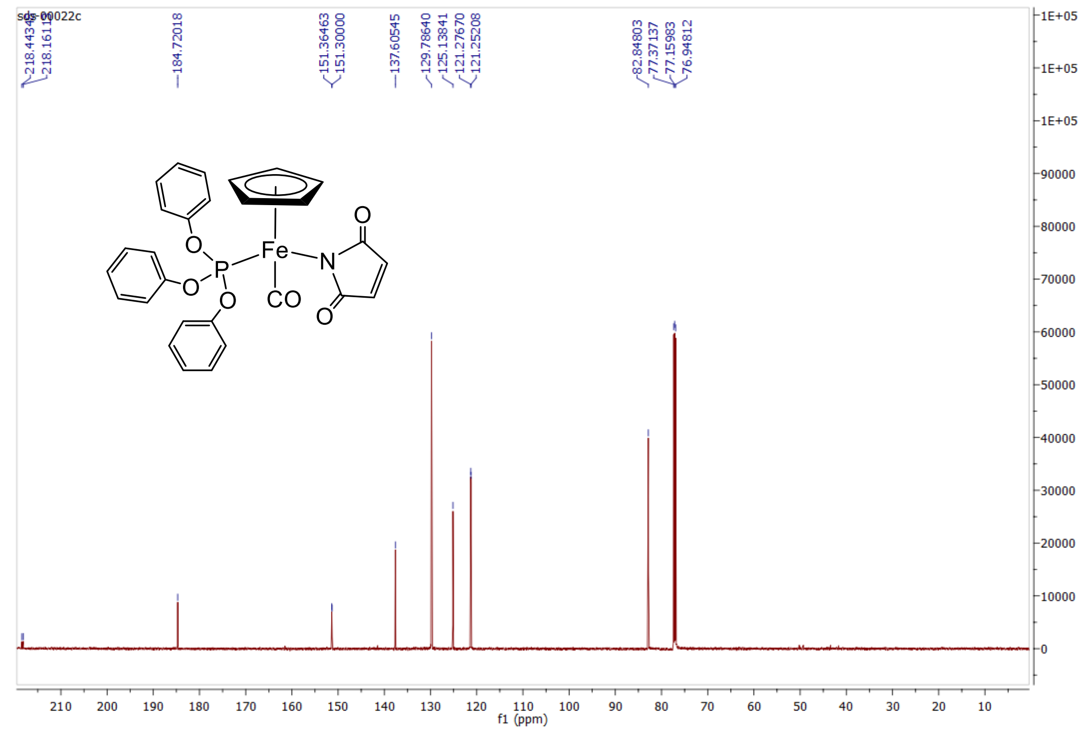

Figure S16. ^{13}C NMR spectra of **3b** in $CDCl_3$.

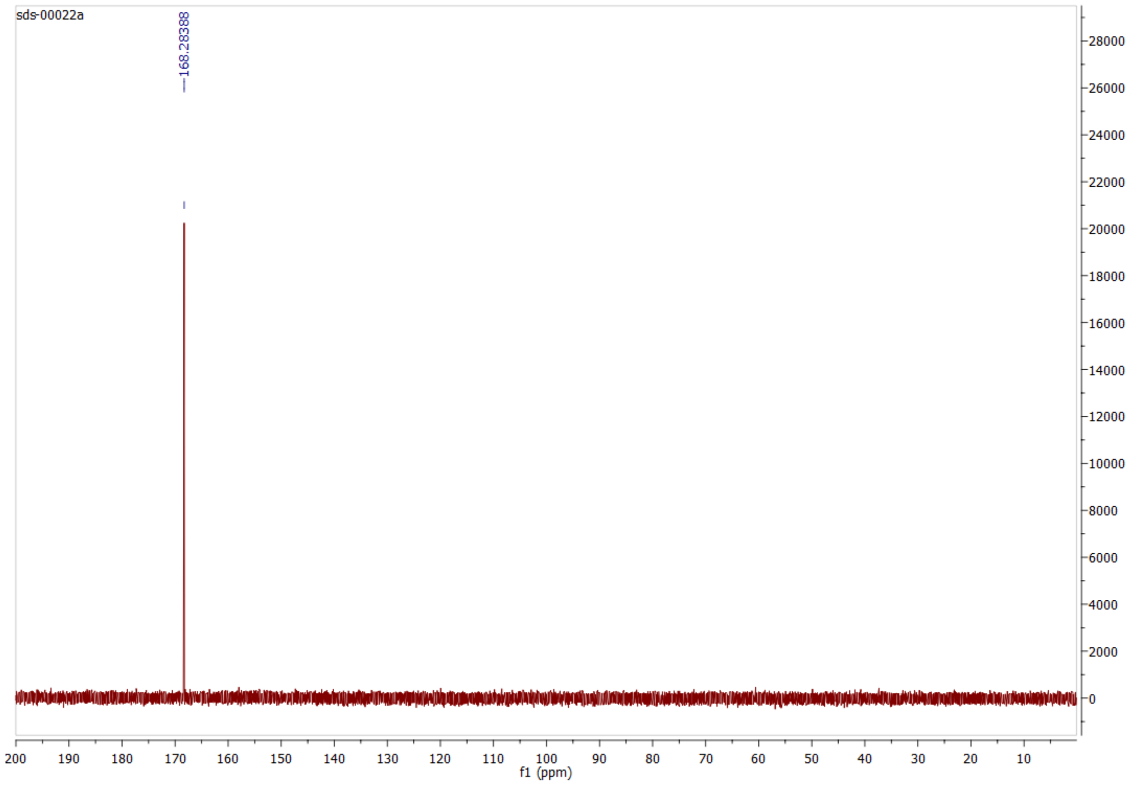

Figure S17. ^{31}P NMR spectra of **3b** in $CDCl_3$.

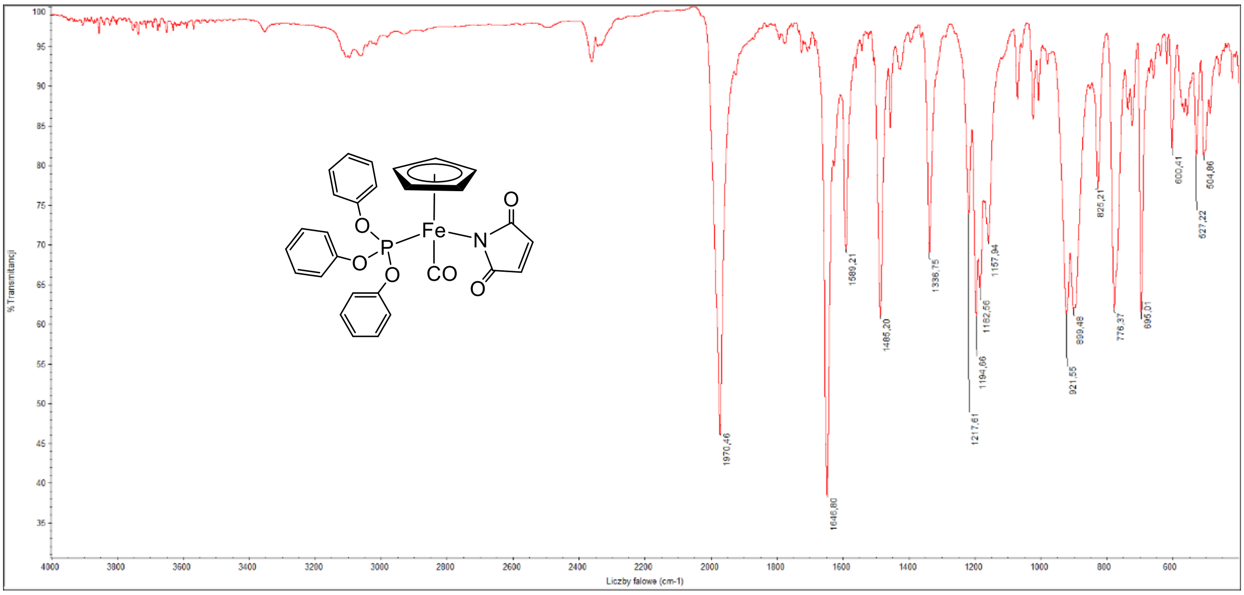

Figure S18. FTIR spectra of **3b**.

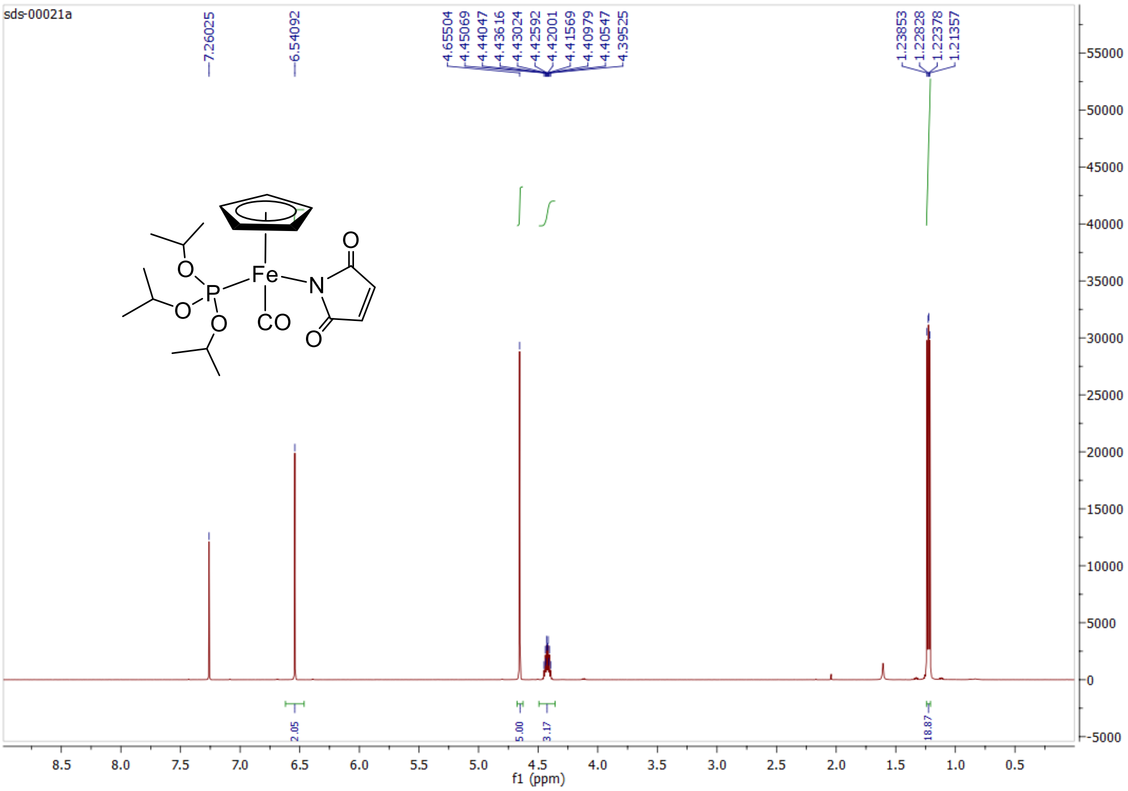

Figure S19. ^{1}H NMR spectra of **3c** in $CDCl_3$.

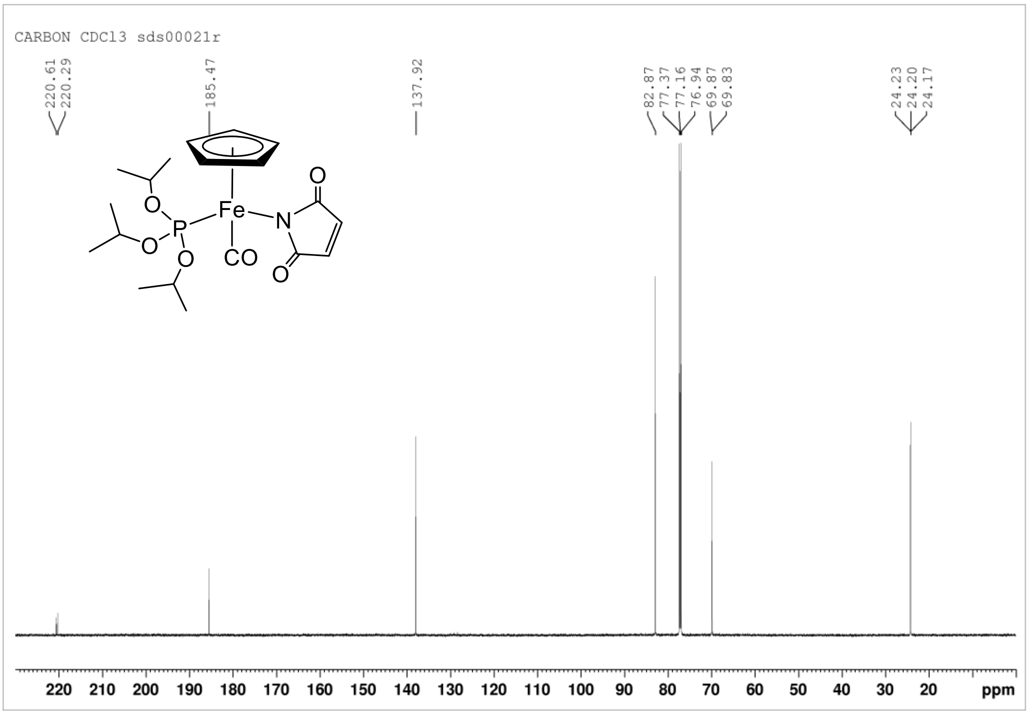

Figure S20. ^{13}C NMR spectra of **3c** in $CDCl_3$.

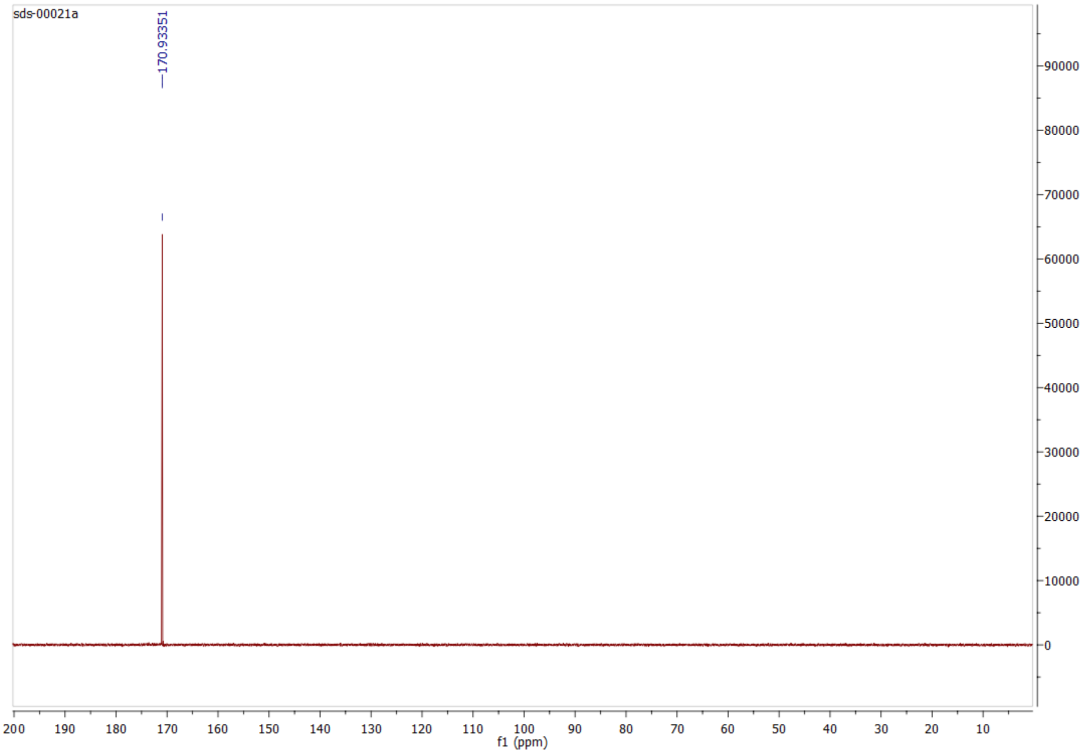

Figure S21. ^{31}P NMR spectra of **3c** in $CDCl_3$.

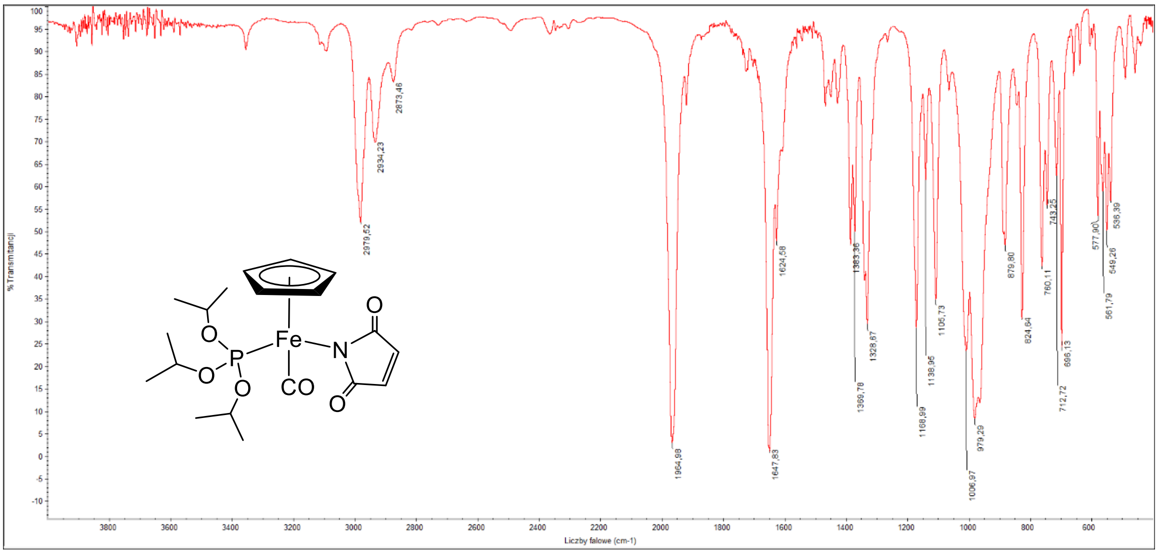

Figure S22. FTIR spectra of **3c**.

2. Crystallographic analysis and data:

Table S1. Selected bond lengths [Å] and angles [°].

	**2a**	**2b**	**3b**	**3c**
Fe1-P1	2.221(2)	2.176(2)	2.144(4)	2.153(4)
Fe1-N1	1.976(2)	1.965(4)	1.976(2)	1.973(2)
Fe1-C10	1.754(2)	1.773(5)	1.761(2)	1.760(2)
Fe1-Cg1 (A / B)	1.7174(7)	1.720(2)	1.744(4) / 1.713(5)	1.719(2)
O10-C10	1.150(2)	1.139(6)	1.149(2)	1.152(2)
P1-C21 / O20	1.829(2)	1.815(4)	1. 622(2)	1.601(2)
P1-C31 / O30	1.837(2)	1.810(4)	1. 605(2)	1.591(2)
P1-C41 / O40	1.833(2)	1.808(4)	1.601(2)	1.596(2)
N1-C2	1.376(2)	1.375(6)	1.378(2)	1.381(2)
N1-C5	1.382(2)	1.380(6)	1.380(2)	1.379(2)
O2-C2	1.223(2)	1.213(6)	1.224(2)	1.219(2)
O5-C5	1.222(2)	1.213(6)	1.220(2)	1.223(2)
N1-Fe1-P1	94.2(2)	94.7(2)	91.9(3)	95.8(2)
N1-Fe1-C10	92.6(2)	92.6(2)	94.4(2)	95.0(3)
P1-Fe1-C10	90.3(2)	90.1(2)	94.8(2)	91.3(5)
N1-Fe1-Cg1 (A / B)	121.8(1)	120.8(1)	115.8(1) / 115.0(1)	122.6(1)
P1-Fe1-Cg1 (A / B)	126.0(1)	124.8(1)	124.9(1) / 125.3(1)	121.3(1)
C10-Fe1-Cg1 (A / B)	122.9(1)	125.0(1)	126.5(1) / 116.4(1)	123.1(1)

N1-Fe1-Cg1 (A / B)	121.8(4)	120.8(2)	115.9(2) / 126.1(2)	122.6(5)
P1-Fe1-Cg1 (A / B)	126.0(3)	124.8(2)	124.9(2) / 125.4(2)	121.3(3)
C10-Fe1-Cg1 (A / B)	122.9(5)	125.0(2)	126.5(2) / 116.4(2)	123.1(6)
C21 / O20-P1-Fe1	114.6(2)	122.6(2)	119.1(2)	104.9(4)
C31 / O30-P1-Fe1	117.2(2)	116.9(2)	119.0(2)	119.6(4)
C41 / O40-P1-Fe1	114.1(2)	110.8(2)	113.7(2)	122.2(2)
O20-P1-O30	-	-	97.5(2)	102.8(2)
O20-P1-O40	-	-	103.9(2)	106.3(2)
O30-P1-O40	-	-	100.6(2)	99.1(6)
Fe1-C10-O10	176.0(2)	177.8(4)	174.5(2)	173.9(2)
Fe1-N1-C2	128.8(3)	123.3(3)	124.8(2)	126.2(2)
Fe1-N1-C5	123.6(2)	125.3(3)	124.8(3)	126.0(2)
N1-C2-C3	108.5(2)	108.1(4)	108.7(2)	108.4(2)
N1-C5-C4	108.5(2)	107.9(4)	108.7(2)	108.8(2)
N1-C2-O2	126.6(2)	127.2(5)	125.9(2)	126.1(2)
N1-C5-O5	126.2(2)	126.2(4)	125.7(2)	126.6(2)
C2-N1-C5	107.6 (3)	108.7(4)	107.4(2)	107.7(2)
O2-C2-C3	124.9(3)	124.6(4)	125.5(2)	125.5(2)
O5-C5-C4	125.2(2)	125.9(4)	125.6(2)	124.6(2)
N1-Fe1-P1-C21/ O20	13.93(6)	33.7(2)	-137.6(1)	149.0(1)
N1-Fe1-P1-C31 / O30	-111.3(1)	150.0(1)	-18.9(1)	-96.5(1)
N1-Fe1-P1-C41 / O40	130.2(1)	-88.3(2)	99.4(1)	28.4(1)
C10-Fe1-P1-C21 / O20	-78.7(1)	-58.9(2)	-43.0(1)	53.9(1)
C10-Fe1-P1-C31 / O30	156.1(1)	57.4(2)	75.7(1)	168.3(1)
C10-Fe1-P1-C41 / O40	37.6(1)	179.1(2)	-166.0(1)	-66.8(1)
C2-N1-Fe1-P1	52.0(1)	-138.8(3)	126.1(1)	60.5(1)
C5-N1-Fe1-P1	-34.5(1)	152.5(4)	-63.6(1)	-124.5(1)

*Cg1 corresponds to the centre of gravity of cyclopentadienyl ring.

Table S2. Geometric parameters of selected C-H…O hydrogen bonds – distances [Å] and angles [°].

hydrogen bond	D-H	H…A	D…A	< D-H…A	symmetry
**2a**					
C4-H4…O10	0.95	2.44	3.379(1)	172	1-x,1-y,1-z
C13-H13…O10	0.95	2.47	3.361(1)	156	1-x,-y,1-z
C43-H43…O5	0.95	2.61	3.539(1)	167	1-x,-y,1-z
C11-H11…O5	0.95	2.64	3.516(1)	154	-x,1-y,1-z
C44-H44…O2	0.95	2.64	3.576(1)	167	1+x,-1+y,z
**2b**					
C11-H11…O10	0.95	2.49	3.387(5)	158	-x,-y,1/2+z
C43-H43…O2	0.95	2.63	3.144(6)	115	x,1+y,z
C14-H14…O5	0.95	2.27	3.211(6)	172	-x,-y,-1/2+z
**3b**					
C14B-H14B…O10	0.95	2.67	3.335(1)	153	1/2-x,3/2-y,-z

C33-H33…O5	0.95	2.65	3.235(1)	165	1/2+x,1/2+y,z
C34-H34…O5	0.95	2.51	3.165(2)	126	1/2+x,1/2+y,z
C44-H44…O5	0.95	2.56	3.371(2)	144	1/2-x,-1/2+y,1/2-z
C22-H22…O2	0.95	2.55	3.210(1)	127	x,-1+y,z
C12B-H12B…O2	0.95	2.52	3.396(1)	153	x,-1+y,z
**3c**					
C23-H23B…O10	0.98	2.63	3.425(1)	139	1-x,1/2+y,1/2-z
C32-H32B…O5	0.98	2.67	4.175(1)	147	x,1/2-y,1/2+z
C33-H33B…O5	0.98	2.55	3.435(1)	149	x,1/2-y,1/2+z
C13-H13…O2	0.95	2.66	3.403(1)	131	x,1/2-y,-1/2+z

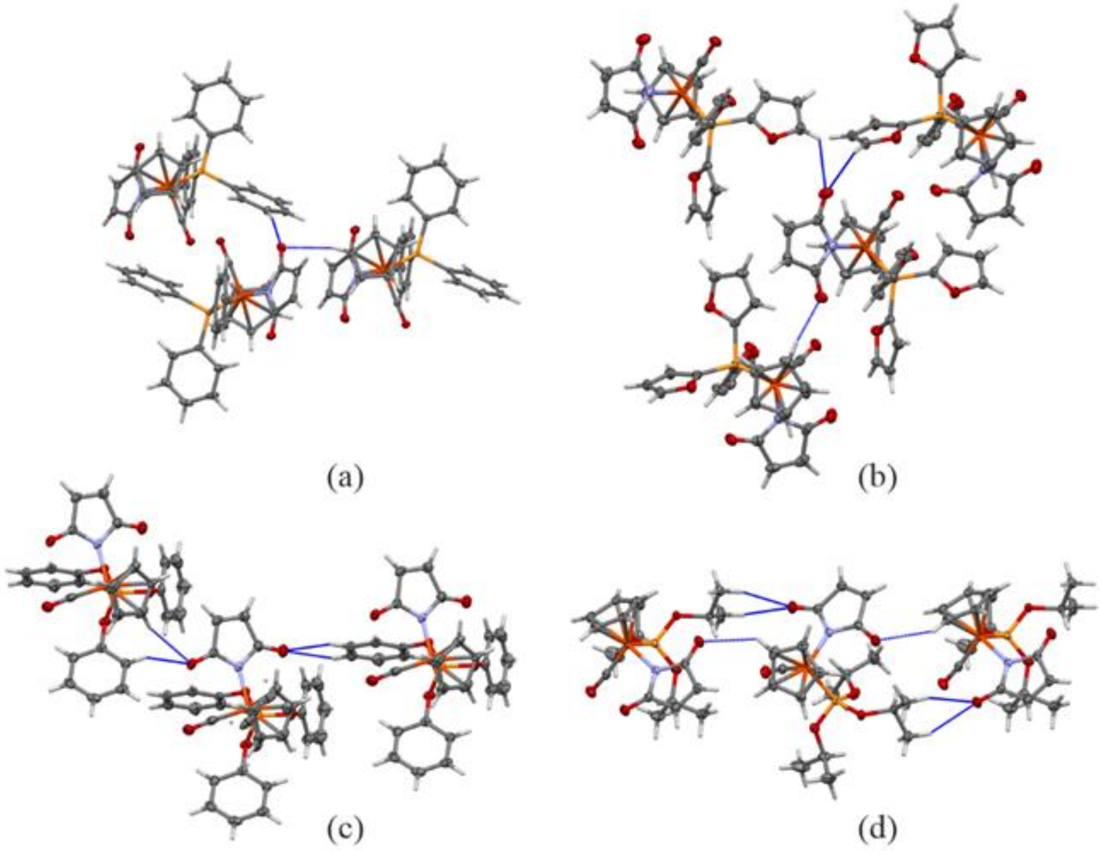

Figure S23. Schemes of selected C-H…O=C hydrogen bonds in the crystal structures: **2a** (a), **2b** (b), **3b** (c) and **3c** (d).

3. Comparative absorbance and emission study:

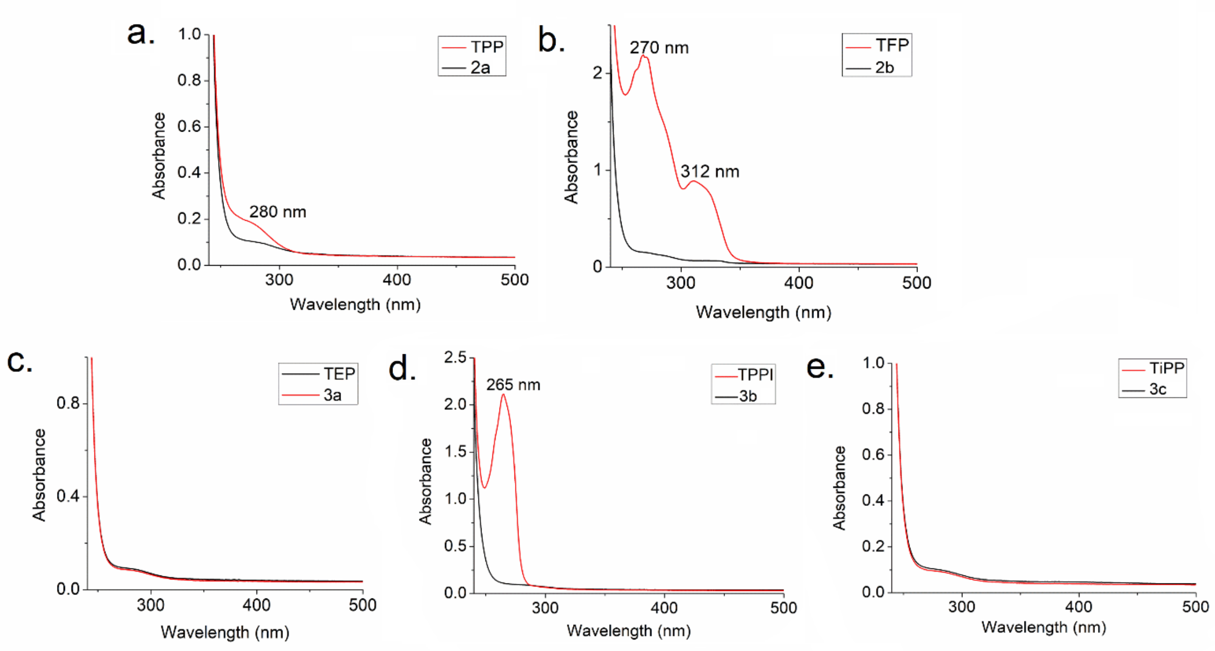

Figure S24. Comparative absorbance spectra of the complexes **2a**, **2b**, **3a**, **3b** and **3c** with respect to corresponding ligands in chloroform. TPP: triphenylphosphine; TFP: tri(2-furyl) phosphine; TEP: triethyl phosphite; TPPI: triphenyl phosphite; TiPP: triisopropyl phosphite.

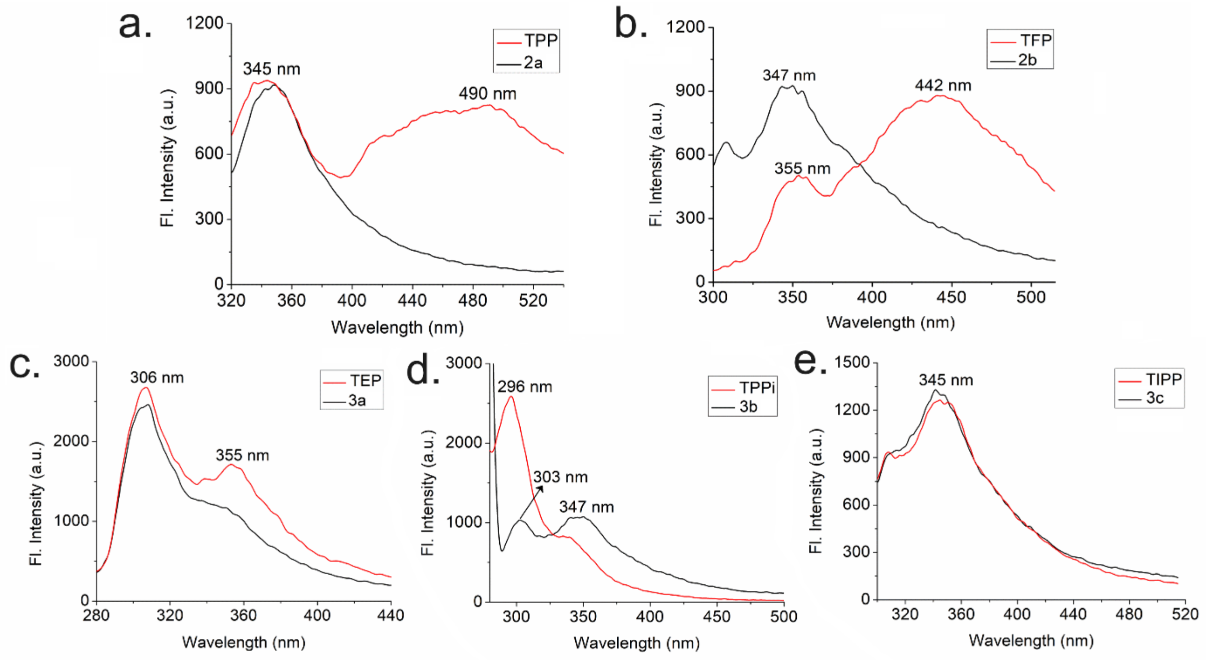

Figure S25. Comparative emission spectra of the complexes **2a**, **2b**, **3a**, **3b** and **3c** with respect to corresponding ligands in chloroform. TPP: triphenylphosphine; TFP: tri(2-furyl) phosphine; TEP:

triethyl phosphite; TPPI: triphenyl phosphite; TiPP: triisopropyl phosphite. The excitations were recorded at the following wavelengths: a) 300 nm, b) 280 nm, c) 260 nm, d) 270 nm and e) 280 nm.

4. Biological studies

Table S3. The viability of PBM, HL-60 and A549 cells after 2 h incubation with iron(II) complexes. The viability for individual samples were calculated relative to control ± SD.

Concentration (μM)	1	2a	2b	3a	3b	3c
**PBM cells**						
0.5	101.07 ± 0.78	98.55 ± 3.27	98.07 ± 0.32	83.62 ± 1.79***↓	98.5 ± 2.18	97.72 ± 1.53
1	100.74 ± 0.73	98.65 ± 4.06	97.62 ± 0.66	82.59 ± 3.38***↓	99.88 ± 2.57	98.2 ± 1.54
2.5	99.56 ± 1.96	98.07 ± 3.18	96.06 ± 2.38	83.52 ± 1.8***↓	97.35 ± 5.51	97.72 ± 2.95
5	99.26 ± 2.82	98.94 ± 2.53	97 ± 1.29	86.67 ± 4.18***↓	100.32 ± 3.02	101.01 ± 3.16
10	101.49 ± 4.25	101.13 ± 3.14	96.53 ± 1.24	89.89 ± 2.91***↓	99.06 ± 3.6	107.01 ± 1.15***↑
25	99.86 ± 1.87	102.46 ± 3.1	93.16 ± 4.6**↓	98.19 ± 4.2	98.42 ± 1.87	113.38 ± 3.02***↑
50	100.66 ± 0.97	102.7 ± 2.19	79.21 ± 1.4***↓	98.5 ± 3.37	100.03 ± 0.88	114.88 ± 2.37***↑
100	100.59 ± 1.01	104.68 ± 1.6**↑	78.49 ± 1.24***↓	97.79 ± 4.89	98.48 ± 0.54	110.29 ± 4.8***↑
250	93.17 ± 2.92***↓	104.85 ± 1.37**↑	75.23 ± 0.62***↓	98.17 ± 2.87	99.51 ± 1.24	9.06 ± 3.64 ***↓
**HL-60 cells**						
0.5	101.68 ± 0.96	104.08 ± 2.11**↑	106.51 ± 2.33**↑	103.48 ± 2.11	108.58 ± 5.93	107.1 ± 1.14***↑
1	101.06 ± 1.48	103.34 ± 3.15*↑	106.74 ± 1.35**↑	105.44 ± 1	105.27 ± 5.97	104.09 ± 3.32
2.5	100.83 ± 2.04	103.43 ± 2.81*↑	103.04 ± 1.98*↑	102.35 ± 0.54	111.13 ± 4.29**↑	108.13 ± 0.47***↑
5	97.79 ± 1.25*↓	108.69 ± 1.98***↑	104.72 ± 2.62**↑	100.13 ± 1.86	116.45 ± 4.31***↑	110.56 ± 8.23
10	89.74 ± 2.7***↓	115.82 ± 2.26***↑	104.03 ± 2.08**↑	101.73 ± 3.47	125.67 ± 4.5***↑	118.49 ± 3.46***↑
25	83.96 ± 1.62***↓	120.78 ± 3.7***↑	95.86 ± 2.66**↓	97.68 ± 2.4	140.68 ± 7.01***↑	126.91 ± 3.29***↑
50	73.55 ± 0.27***↓	118.68 ± 5.5***↑	90.45 ± 0.98***↓	93.65 ± 1.73**↓	116.21 ± 2.09***↑	133.68 ± 3.55***↑
100	24.73 ± 1.89***↓	116.98 ± 3.65***↑	76.15 ± 3.1 ***↓	87.54 ± 2.53***↓	70.87 ± 2.53***↓	138.39 ± 6.32***↑
250	15.71 ± 0.21***↓	113.31 ± 3.08***↑	21.23 ± 1.56 ***↓	30.8 ± 1.55***↓	23.24 ± 0.25 ***↓	34.32 ± 1.58***↓
**A549 cells**						
0.5	93.79 ± 10.65	84.75 ± 11.62*↓	84.30 ± 11.24	100.11 ± 13.46	94.42 ± 12.46	95.70 ± 6.80
1	93.56 ± 10.37	71.98 ± 10.47***↓	80.74 ± 14*↓	89.02 ± 8.33	97.15 ± 8.30	87.36 ± 5.46
2.5	78.45 ± 9.98**↓	67.99 ± 8.45***↓	82.33 ± 9.73	86.36 ± 7.43*↓	96.59 ± 9.16	96.70 ± 14.69
5	67.97±12.88***↓	63.83 ± 8.57***↓	83.32 ± 11.51	85.49 ± 7.39*↓	90.46 ± 7.14	98.37 ± 10.99
10	63.34 ± 13***↓	64.49 ± 7.71***↓	101.28 ± 15.65	83.61 ± 12.73**↓	93.75 ± 11.55	102.84 ± 12.35
25	51.29 ± 9.65***↓	64.05 ± 10.66***↓	114.98 ± 11.35	82.29 ± 5.9***↓	97.77 ± 7.39	122.34 ± 18.78*↑
50	41.64 ± 8.89***↓	71.32 ± 8.08***↓	123.09 ± 7.89**↑	82.69 ± 3.89**↓	104.82 ± 7.84	124.75 ± 9.61*↑
100	36.49 ± 6.19***↓	68.25 ± 7.97***↓	84.95 ± 8.31	74.97 ± 6.81***↓	97.69 ± 11.65	122.01 ± 9.34
250	36.35 ± 4.86***↓	57.40 ± 11.79***↓	40.75 ± 4.24***↓	68.50 ± 8.56***↓	97.95 ± 13.58	134.59±21.57***↑

Cell viability in the control was taken as 100%. * $p < 0.05$, ** $p < 0.01$, *** $p < 0.001$, ↑ − increase, ↓ − decrease

Table S4. The viability of PBM. HL-60 and A549 cells after 24 h incubation with iron(II) complexes. The viability for individual samples were calculated relative to control ± SD.

Concentration (μM)	1	2a	2b	3a	3b	3c
**PBM cells**						
0.5	106.05 ± 1.67***↑	101.47 ± 3.95	105.37 ± 7.62	102.49 ± 0.39	97.8 ± 2.01	99.43 ± 2.93
1	103.84 ± 0.59***↑	98.54 ± 1.78	101.81 ± 0.86	104.14 ± 1.97***↑	98.46 ± 7.18	101.13 ± 1.2
2.5	99.3 ± 1.2	101.97 ± 3.08	97.1 ± 1.83	104.31 ± 0.56***↑	94.41 ± 2.27**↓	100.22 ± 2.17
5	91.84 ± 1.72***↓	102.32 ± 1.76	94.2 ± 4.56**↓	102.43 ± 1.43	52.91 ± 0.63***↓	121.77 ± 1.7***↑
10	85.39 ± 1.19***↓	88.22 ± 1.4***↓	94.94 ± 2.09**↓	99.21 ± 0.78	49.35 ± 1.94***↓	104.73 ± 5.85
25	89.23 ± 0.44***↓	64.32 ± 0.17***↓	88.93 ± 2.18***↓	98.34 ± 0.97	31 ± 1.1***↓	87.47 ± 3.78***↓
50	78.87 ± 1.76***↓	55.37 ± 0.71***↓	85.34 ± 2.58***↓	98.58 ± 2.84	28.33 ± 2.17***↓	86.55 ± 1.9***↓
100	50.82 ± 1.08***↓	38.14 ± 0.33***↓	83.38 ± 3.4***↓	102.37 ± 3.3	16.65 ± 0.55***↓	59.71 ± 6.05***↓
250	21.96 ± 0.71***↓	32.79 ± 1.16***↓	77.59 ± 2.06***↓	94.72 ± 3.88**↓	13.82 ± 0.52***↓	1.7 ± 0.33***↓
**HL-60 cells**						
0.5	106.53 ± 5.45	103.09 ± 2.54	107.57 ± 3.87**↑	107.33 ± 1.24***↑	101.68 ± 1.04	104.58 ± 2.43**↑
1	107.4 ± 3.25**↑	104.21 ± 1.11**↑	105.5 ± 3.13*↑	109.38 ± 4***↑	98.91 ± 4.85	104.15 ± 1.69**↑
2.5	109.43 ± 1.41***↑	100.69 ± 4.9	97.63 ± 0.5**↓	112.36 ± 1.5***↑	91.42 ± 3.16***↓	104.77 ± 2.86**↑
5	89.27 ± 2.42***↓	79.99 ± 2.5***↓	79.34 ± 1.21***↓	106.89 ± 3.43*↑	72.37 ± 3.19***↓	92.44 ± 1.36***↓
10	42.91 ± 0.62***↓	52.57 ± 0.94***↓	51.6 ± 1.43***↓	89 ± 3.39***↓	49.27 ± 1.71***↓	61 ± 0.47***↓
25	37.45 ± 1.48***↓	15.3 ± 1.21***↓	38.21 ± 0.22***↓	46.28 ± 0.82***↓	3.18 ± 0.36***↓	43.39 ± 0.59***↓
50	30.99 ± 0.27***↓	10.1 ± 0.23***↓	35.8 ± 2.09***↓	38.62 ± 0.52***↓	0.99 ± 0.02***↓	38.16 ± 0.74***↓
100	14.21 ± 0.37***↓	10.31 ± 0.23***↓	25.41 ± 1.92***↓	39.69 ± 0.83***↓	0.96 ± 0.1***↓	10.26 ± 1.19***↓
250	3.83 ± 0.21***↓	8.4 ± 0.12***↓	17.52 ± 1.12***↓	17.5 ± 0.5***↓	0.94 ± 0.06***↓	1.08 ± 0.06***↓
**A549 cells**						
0.5	75.31 ± 7.67**↓	73.24 ± 14.30*↓	81.57 ± 14.09	80.91 ± 12.58	91.38 ± 8.55	84.79 ± 18.99
1	70.02 ± 8.73***↓	63.60 ± 12.84***↓	74.40 ± 19.47*↓	70.97 ± 10.92*↓	92.49 ± 11.20	73.25 ± 14.25**↓
2.5	73.33 ± 7.65**↓	68.69 ± 13.05**↓	73.54 ± 23.46*↓	68.70 ± 18.12*↓	78.64 ± 6.01***↓	65.38 ± 12.33***↓
5	104.37 ± 10.70	65.32 ± 9.49**↓	61.87 ± 14.65***↓	68.55 ± 13.94*↓	80.46 ± 7.49***↓	69.03 ± 8.49***↓
10	108.72 ± 15.90	114.65 ± 23.41	87.91 ± 9.79	71.63 ± 17.67*↓	80.04 ± 11.40***↓	103.54 ± 13.76
25	82.97 ± 8.04	117.99 ± 19.59	110.30 ± 11.49	131.92 ± 19.26*↑	25.66 ± 3.11***↓	105.27 ± 11.18
50	50.31 ± 12.68***↓	105.46 ± 17.32	91.31 ± 16.37	131.87 ± 25.64*↑	22.34 ± 2.60***↓	88.99 ± 15.62
100	9.43 ± 1.69***↓	120.77 ± 9.93	13.33 ± 3.51***↓	153.54 ± 19.26***↑	42.71 ± 5.87***↓	75.00 ± 9.56**↓
250	2.35 ± 0.49***↓	90.11 ± 9.87	1.39 ± 0.12***↓	95.62 ± 13.21	28.61 ± 5.09***↓	87.20 ± 10.62

Cell viability in the control was taken as 100%. * $p < 0.05$, ** $p < 0.01$, *** $p < 0.001$, ↑ − increase, ↓ − decrease

5. Molecular docking studies

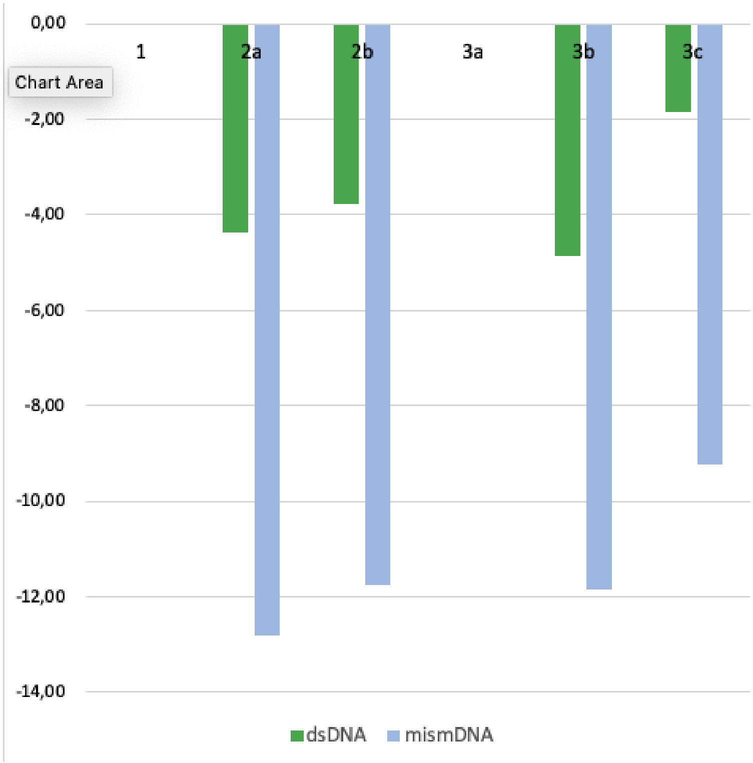

Figure S26. The binding energy values [kcal/mol] obtained by molecular docking of tested compounds **2a-b**. **3b-c** with fully paired ds-DNA and mismatched DNA.

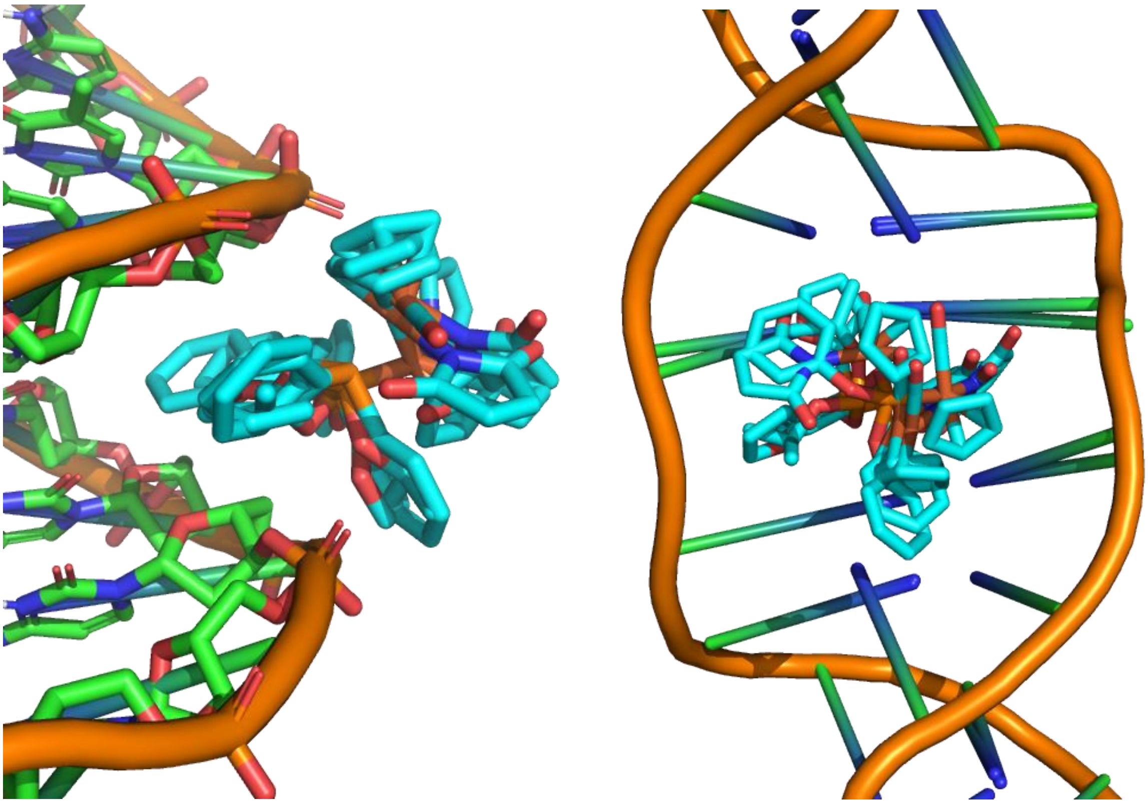

a **b**

Figure S27. The view of structures for (a) fully paired 12nt ds-DNA fragment will all tested compounds located in minor groove of DNA and (b) mismatched 12nt DNA fragment (T-T mismatch) with all tested compounds located at the level of the mismatch.
